# Acute effect of light and time of day on thermal physiology, perception, and behavior

**DOI:** 10.1038/s41598-025-22542-w

**Published:** 2025-11-04

**Authors:** Abdelkader Elkounni, Marika Vellei, Jérôme Le Dréau, Marcel Schweiker, Christian Inard

**Affiliations:** 1 Laboratory of Engineering Sciences for the Environment LaSIE UMR CNRS 7356, La Rochelle University, 17000 La Rochelle, France; 2 University of Bordeaux, CNRS, Bordeaux INP, I2M,UMR 5295, F-33400 Talence, France; 3 Arts et Metiers Institute of Technology, CNRS, Bordeaux INP, I2M,UMR 5295, F-33400 Talence, France; 4 Healthy Living Spaces Lab, Medical Faculty, Institute for Occupational, Social and Environmental Medicine, RWTH Aachen University, Pauwelsstr. 30, 52074 Aachen, Germany; 5 Chair of Healthy Living Spaces, Faculty of Architecture, RWTH Aachen University, Pauwelsstr. 30, 52074 Aachen, Germany

**Keywords:** Neuroscience, Physiology

## Abstract

Implementing novel, human-centric building control strategies that account for the interaction of multidomain factors, light, temperature, and time of day, can enhance occupant comfort and promote energy savings. However, their successful implementations require a robust framework that clearly explains the physiological mechanisms through which light exposure influences thermal perception and evaluation. To date, most studies have primarily relied on the hue-heat hypothesis, which attributes changes in thermal assessment solely to visual color associations, overlooking the physiological impact of light, particularly blue-enriched bright light, on the circadian rhythm and thermoregulation. In this study, we investigated the influence of light intensity on thermal physiology, perception, and behavior within a circadian context, employing skin temperature measurements, subjective questionnaires, and fan-use behavior as outcome measures. 20 healthy adults participated in four experimental sessions combining two illuminance levels (bright vs. dim, with identical spectral composition) at two times of day (07:00 and 14:00) under warm conditions, both steady-state and fan-induced dynamic ones. Results showed that skin temperature followed its natural circadian rhythm, being lower in the early morning than at midday. Notably, bright light exposure significantly suppressed the circadian rise in skin temperature in the morning, shifted thermal sensation votes from slightly warm towards neutral, and improved thermal comfort votes compared to dim light or midday exposure. This effect was evident under steady-state warm conditions and persisted following fan use. In contrast, under the fan-induced slightly cool condition, neither light intensity nor time of day significantly affected thermal assessment. These findings underscore the fundamental role of circadian physiology in thermal comfort and suggest that strategically timed light and thermal exposure can optimize comfort by aligning ambient conditions with the body’s internal rhythms.

## Introduction

### Context

Humans are continuously exposed to a mix of sensory inputs, whether indoors or outdoors, experiencing simultaneously auditory, olfactory, visual, and thermal stimuli^1^. These combined sensory inputs significantly shape individuals’ overall comfort and satisfaction with their physical surroundings^2^. In recent years, there has been growing interest in cross-modal influences from one modality of perception to another^3,4^, with a particular focus on how light affects thermal perception and evaluation. Various reviews^5–8^ have provided continuous updates on the influences of light on subjective thermal assessment (e.g., thermal sensation, comfort, acceptability, preference) and/or physiological responses (e.g., skin, core body temperature, heart rate). The practical potential of using light exposure as a modulator of subjective thermal responses is promising. By considering the interaction of multidomain factors such as light, temperature, and time of day, novel human-centric building control strategies can be developed to enhance comfort levels and achieve energy savings in buildings^9,10^. However, such implementation requires a well-founded framework that clearly describes the physiological mechanisms through which light exposure influences autonomic thermoeffectors and thermal assessment.

### Background

**Figure 1:**
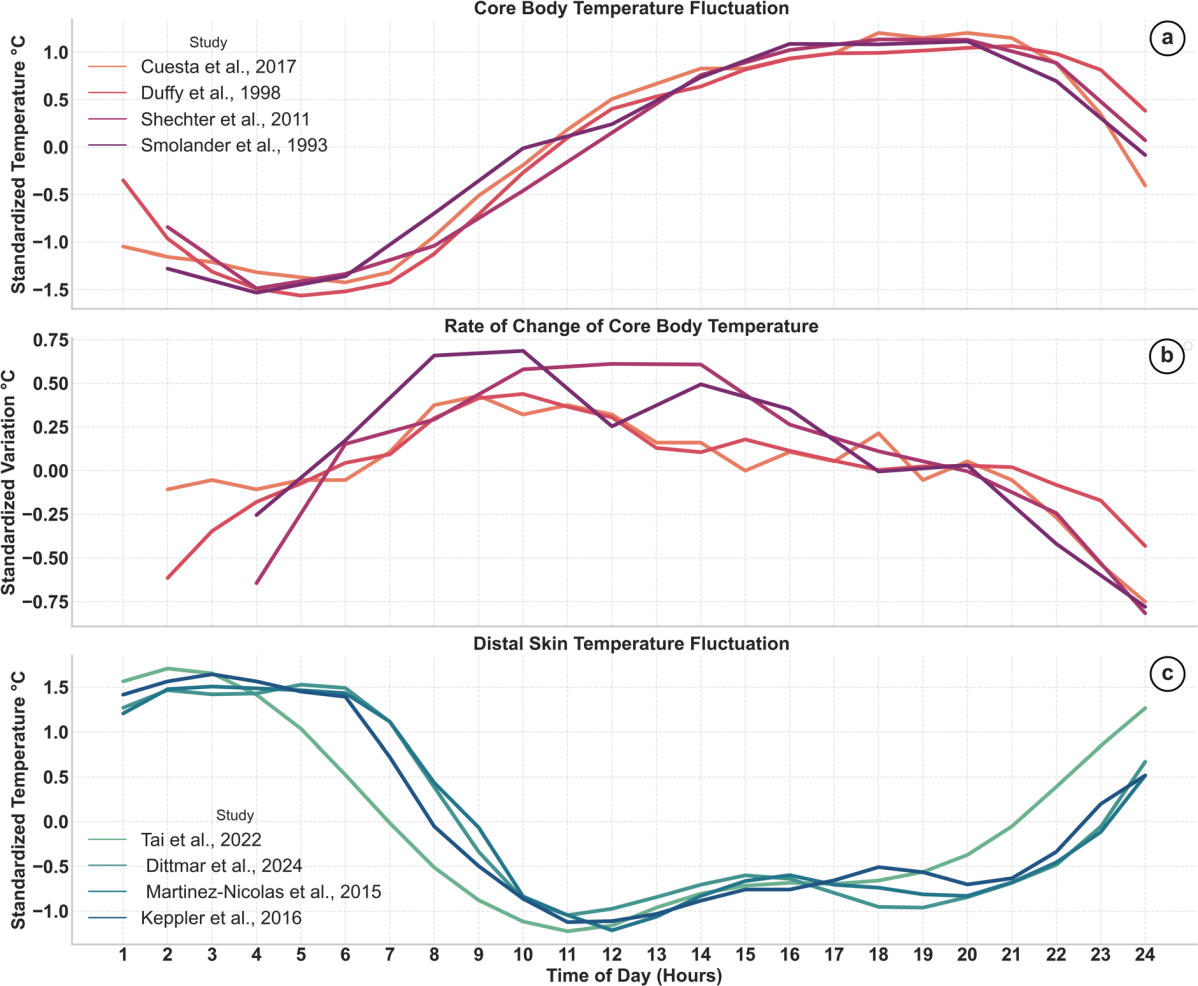
Standardized core body temperature, its rate of change and standardized distal skin temperature throughout the day, adapted from^9^.

Human physiological, behavioural, and cognitive states exhibit a 24-h fluctuation known as the circadian rhythm, derived from the Latin words circa (about) and diem (day)^11^. Such oscillations can be observed in various physiological (circadian) markers, including plasma melatonin levels, skin temperature, and core body temperature^12^. For instance, Fig. 1a, b illustrate the diurnal fluctuations in core body temperature and its rate of change, as reported by several studies^13–16^. In contrast, Fig. 1c depicts changes in distal skin temperature over the course of 24 h, as reported in^17–20^. The observed rhythm in body temperature is primarily driven by variations in heat production and heat loss throughout the day^21^. During sleep initiation, an increase in heat dissipation occurs through distal vasodilation, leading to elevated distal skin temperature, alongside a decrease in metabolic heat production^22^. These changes result in a decline in core body temperature, which is essential for facilitating the onset of deep sleep. In contrast, during the awake state in the morning, distal skin temperature decreases rapidly, indicating vasoconstriction, while heat production increases gradually. These combined effects result in a rise in core body temperature throughout the day^23^, as shown in Fig. 1a, c.

The 24-h pattern of core body temperature, along with other circadian markers, is primarily regulated by the body’s internal clock, which synchronises to external time cues (e.g., light) to ensure optimal physiological and behavioural responses to the environment^24^. Light is considered a predominant zeitgeber (time giver) and plays a significant role in entrainment, i.e., the process by which the internal biological clock aligns and synchronises its period with external stimuli^25^. In addition to light, other factors such as feeding behaviour, social timing, and physical activity (non-photic zeitgebers) also contribute to circadian entrainment^26^. For the internal clock to entrain and maintain a certain period and phase of the circadian rhythm, a sustained and consistent exposure to zeitgebers is required^27^. In contrast, brief exposures to an exogenous stimulus superimposed on an established state of entrainment can cause attenuation or enhancement of a circadian marker of interest^28^. Chronobiologists refer to these acute effects as masking, indicating the direct and immediate impact external stimuli exert on the biological clock^29^. Such masking effects are observed with abrupt, brief (i.e., acute) exposure to light, particularly in neuroendocrine responses^30^. For example, a single 12-min bright light pulse is sufficient to induce a shift in the human pacemaker^31^. A given state of entrainment is largely influenced by the timing of light exposure (time of day). A phase response curve quantifies both the magnitude and directionality of circadian phase shifts (advance/delay) elicited by light at different times of day^32–34^. In diurnal species, positive masking occurs during the day, when exposure to bright light increases core body temperature, which is already elevated in alignment with the endogenous circadian drive for wakefulness. Negative masking occurs during the night when light suppresses melatonin secretion, overriding the circadian pacemaker’s sleep-promoting signals^35^. Thus, masking responses are also phase-dependent, reflecting either amplification (positive masking) or suppression (negative masking) of physiological processes relative to the internal circadian phase.

Light properties are important in masking and entrainment. For example daylight, under which the internal clock is typically entrained, has two main characteristics: high illuminance, reaching up to 100,000 lx under direct sunlight and 10,000 to 25,000 lx in daylight conditions^36^ , and a high energy content in the blue region of the visible spectrum ($\lambda =450-460 \;nm$)^37^. Other factors contribute to the effect of light, including the duration of exposure and light history^30^. For humans, exposure to bright light, especially short-wavelength (blue) radiation in the late evening and early night for 2 h, is sufficient to suppress nocturnal melatonin release, slows the rise in distal skin temperature and inhibits the natural decline in core body temperature^38^, thereby inducing masking of the central temperature rhythm^39,40^.

### State of the art

The existing literature provides a substantial number of controlled experimental studies examining the effect of light on thermal assessment. Within the scope of this work, we have identified and reviewed 34 studies, with a focus on thermal sensation and thermal comfort (see review table in s.1/supplementary Materials for detailed break down). The reviewed literature can be broadly categorized into three types of studies: 1) those that vary lighting color (e.g., warm, cool, red, blue) while keeping intensity constant, with the primary experimental factor being the correlated color temperature (CCT)^41–43^. The range of CCT typically spans from 2000 K to 6500 K; 2) those that vary lighting intensity (e.g., bright, dim) while keeping color constant, where the primary factor is illuminance^3,39,44^, with intensity ranging from 5 lux for dim conditions to 5000 lux for bright conditions; 3) those that manipulate both lighting color and intensity in a factorial design, investigating interactions^45–47^. Among the reviewed studies, the primary quantities of interest include thermal sensation, comfort, and physiological reactions (e.g., heart rate, skin temperature, core body temperature). The most commonly investigated participant category consists of young, healthy adults, with ages typically ranging from 19 to 26 years. Additionally, the samples are generally balanced in terms of sex. Sample sizes vary across studies, with some exceeding 100 participants^48,49^, while earlier studies tend to have smaller sample sizes, 5 to 7 participants^50,51^. In terms of investigated thermal conditions, there are mainly two trends: studies with contrasting thermal conditions (e.g., warm vs. cool)^3,45,52^, and studies with one fixed thermal exposure^53–55^. Our review of 34 studies revealed the following gaps in the existing research:

- Hue-Heat Hypothesis vs Illuminance-Thermal Markers Hypothesis: Rakotoarivelo and Malet-Damour^8^ have identified two primary frameworks for investigating the effects of light on thermal assessment. The first falls under the Hue-Heat Hypothesis, which assumes a certain association between the perceived colour of an object or a light source and the perceived warmth or coolness^56^. The second framework, under the Illuminance-Thermal Markers Hypothesis, suggests that the effects of light on thermal assessment are primarily due to its circadian influence and its ability to regulate the internal body clock. Rakotoarivelo and Malet-Damour^8^ have also highlighted that the Illuminance-Thermal Markers Hypothesis is more widely accepted, with stronger backing from the scientific community compared to the Hue-Heat Hypothesis, which is largely based on psychological associations (beliefs)^57^. 19 of the reviewed studies focus on visual manifestations, such as those described by the Hue-Heat Hypothesis. Accordingly, only 4 studies^43,47,53,54^ have reported melanopic equivalent illuminance (a metric used to determine how much a light source influences the circadian rhythm in humans)^58^, instead relying on traditional metrics like visual illuminance and colour appearance (CCT). However, as described above, light, especially short-wavelength blue light, can acutely alter circadian markers like melatonin and body temperature via masking effects, depending on timing and exposure duration. Given the fundamental role of thermoreceptor activity in the body^59^, both skin temperature and core body temperature play a crucial role in thermal perception and behavioural responses. Consequently, other than its effect on autonomic thermoregulation, light may also influence circadian oscillations in subjective thermal ratings^30^.
- Time of Day Consideration:The effect of light on human thermophysiology is highly dependent on the time of day. However, in the reviewed studies, time of day was not consistently considered in the experimental design or the analysis, and in 19 of reviewed studies, it was not mentioned at all.
- Radiant Gains and Lighting Technology: Light-induced effects, particularly warmer sensations, typically manifest either under high illuminance or enriched blue light settings^49,50,60,61^. High illuminance levels are associated with increased radiant heat gains, especially in earlier studies which often relied on halogen and incandescent lamps. These circumstances explain the pattern of cooler sensations observed under dim light in older studies. Notably, radiant heat contributions were rarely quantified, and radiant temperature was not consistently reported.
- Dynamic Conditions: 28 studies examined the effects of light under steady-state thermal conditions. The influence of light under dynamic thermal conditions remains largely unexplored and warrants further investigation.

### Research question

Building on the research gaps identified in the previous section, this study aims to address the following research question:

**To what extent does the interaction between light and time-of-day alter subjective thermal assessment, and how are these sensory changes reflected in concurrent thermoregulatory physiology and thermal behaviors?**

This question is addressed by investigating the acute effect of light exposure on skin temperature, subjective thermal assessment, and thermoregulatory behaviour at different times of day (early morning and afternoon). The experiment is conducted in a controlled warm environment targeting an ambient air temperature of approximately 30 ${}^{\circ }$C. This moderately warm condition was selected to promote morning rise in core body temperature without causing excessive discomfort or heat strain. Both steady-state and fan-induced dynamic thermal conditions were examined, addressing the predominance of steady-state investigations in the literature.

Core body temperature rises throughout the day, with the peak rate of change occurring in the morning, as illustrated in Fig. 1b. As described above, exposure to bright, blue-enriched light in the morning induces a positive masking effect, promoting an earlier rise through shifts in autonomic thermoregulation (reduced activation of heat dissipation mechanisms). In parallel, thermal assessment and behaviour are expected to align with this physiological trend during the morning, rendering participants more tolerant to warmth, which will subsequently result in a shift towards a cooler sensation. This rationale forms the basis for our first hypothesis:

- *hypothesis 1 :* bright light in the morning will result in a shift in thermal sensation votes toward cooler votes compared to dim light. As a consequence of this first hypothesis, we expect that in terms of thermoregulatory behavior, participants will adjust their fan speed to lower velocities to promote heat gains and allow earlier core body temperature rise.

In the middle of the subjective day, as shown in Fig. 1b, the rate of increase in core body temperature stabilizes and the variation in core body temperature becomes less evident. According to phase response curve^34^, the effect of light is present during this period but with a lower effect compared to the morning. Based on this reasoning, we propose the second hypothesis:

- *hypothesis 2 :* In the middle of the subjective day, different light intensities will not significantly affect occupants’ thermal sensation.

## Methodology

### Experimental design

**Figure 2:**
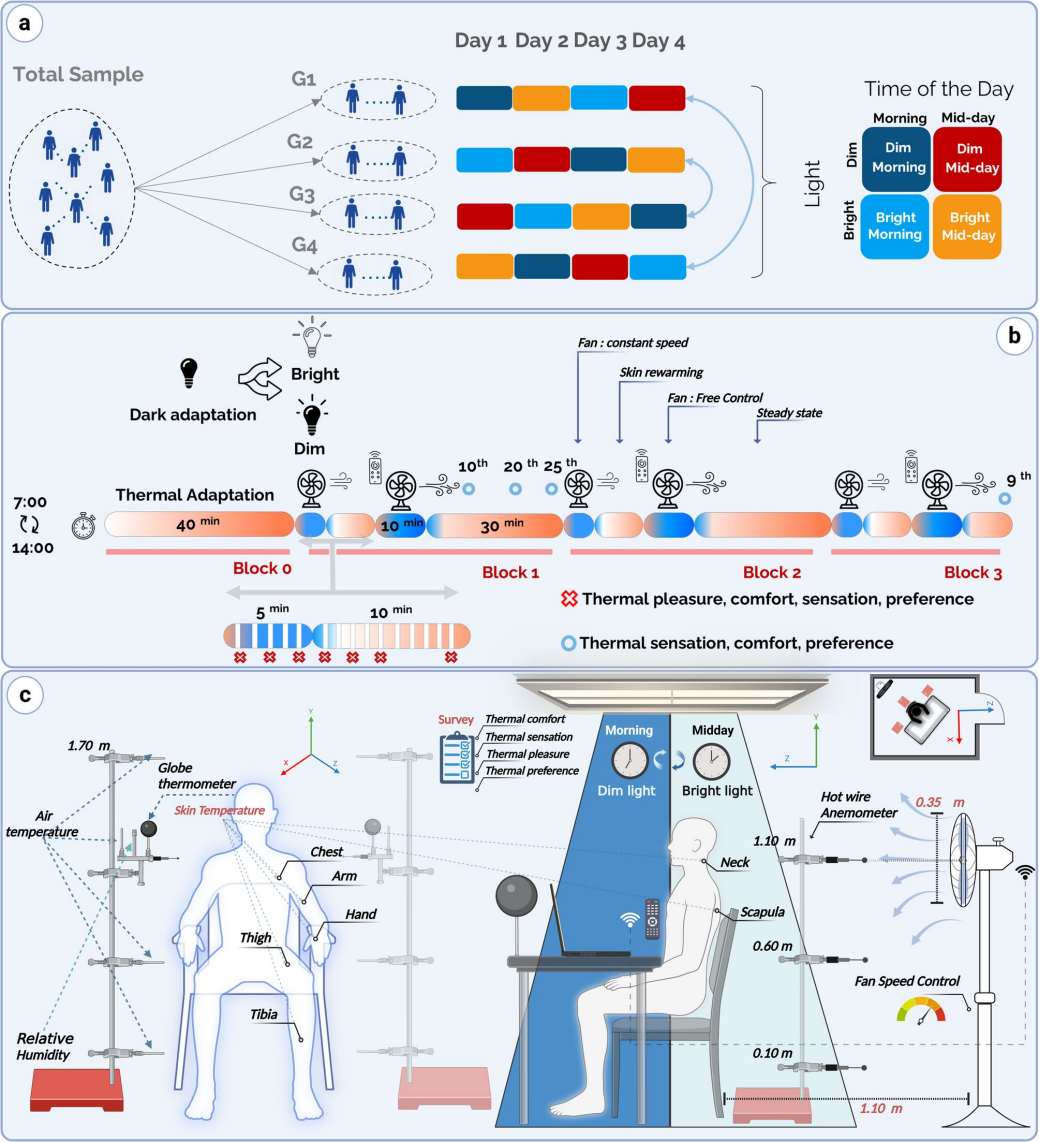
(**a**) Randomization process of exposure conditions across groups. (**b**) Experiment timeline illustrating the different exposure phases: “Fan-at-constant-speed” (5-min sections with the fan icon), “Skin-rewarming” (10-min sections with no fan), “Fan-free-control” (10-min sections with the fan operated via remote control), and “Steady-state condition” (30-min sections with no fan). (**c**) Experimental layout, including environmental and physiological measurements, and subjective thermal assessments.

The proposed design represents a 2 $\times$ 2 factorial design based on two factors with two levels each: time of day (morning 7:00-10:30 vs. midday 14:00-17:30) and light intensity (bright vs. dim), see Fig. 2a. Sensitivity of humans to light is high in the morning (early biological day), with a great potential for positive masking, while it decreases during midday (middle of the biological day)^62^. Although humans exhibit even higher responses to light during the night (biological night), practical constraints (restricted lab accessibility after work hours) made early morning the optimal choice for experimentation.

For the choice of lighting conditions, the rationale is based on the intensity-dependent response of the circadian system to light. Bright light induces a stronger effect on the circadian system, whereas dim light is set as a baseline for comparison due to its relatively smaller effect^63^. In this work, we maintained consistent light color to avoid any confounding effects related to color association under the Hue-Heat Hypothesis. Detailed characteristics and thresholds of the light conditions will be discussed in the light section.

The combination of light and time-of-day factors resulted in four testing conditions: *Bright Light-Morning*, *Bright Light-Midday*, *Dim Light-Morning*, and *Dim Light-Midday*. Each participant experienced one unique condition per day over four consecutive days within the same week. To minimize systematic order effects, the sequence of conditions was counterbalanced across four distinct orders, each assigned to a different subgroup of five participants within the total sample (as illustrated in Fig. 2a).

### Participants

Participants were recruited through a questionnaire distributed at La Rochelle University. The questionnaire was designed to target a homogeneous population, with inclusion criteria focusing on young healthy adults aged 19–30 years and with a Body Mass Index (BMI) between 18.5 and 24.9 kg/m$\phantom{0}^2$, in order to minimize inter-individual variability. Additional criteria included the absence of extreme early or late chronotypes (assessed with the Munich ChronoType Questionnaire, MCTQ ^64^), no jetlag experienced in the week preceding the experiment, and acclimatization to the French climate (at least 1 year of residence in France). Exclusion criteria comprised sickness, excessive missing data, or extreme response patterns (e.g., outlier thermal votes, fan adjustment) during the experimental sessions. Each participant received a compensation of 20 € per hour for their participation. The study involved 20 young, healthy adults, evenly distributed between males and females (1:1 ratio). Table 1 summarizes the participants’ characteristics.

**Table 1:** Participant characteristics presented as mean ± SD.

Characteristics	Men (n = 10)	Women (n = 10)
Age (years)	25.8 ± 2.70	24.0 ± 3.7
Height (m)	1.77 ± 0.07	1.62 ± 0.08
Weight (kg)	72.1 ± 8.40	57.1 ± 6.40
BMI (kg/$\hbox {m}^2$)	22.95 ± 1.77	21.72 ± 1.37
MCTQ (hours:minutes)	4 h 04 m ± 0 h 56 m	4 h 23 m ± 0 h 37 m

The total sample size of 20 was predefined based on prior power calculations for the effect of time-of-day and light interaction on two types of measured outcomes (continuous and ordinal). For the continuous variable skin temperature, we conducted a simulation-based power analysis following the approach of^65,66^. First, we generated an artificial dataset to simulate the anticipated impact of light and time-of-day on skin temperature across a range of effect sizes. This simulated data was then fed into a Gaussian mixed-effects model to estimate the effects of light, time-of-day, and their interaction under the alternative hypothesis. Specifically, we evaluated three standardized effect sizes, small ($\beta = 0.1$), medium ($\beta = 0.3$), and large ($\beta = 0.5$) as categorized by^67^. To account for the repeated-measures design, we adopted a mixed-effects structure that incorporated random intercepts. The variance of these random intercepts ($\sigma$) was set to $\sigma = 1$, 1.5, and 2. The simulation results showed that for a standardized $\beta = 0.5$, the power averaged over tested values of $\sigma$ attain 85% for the selected sample size.

For outcomes measured on an ordinal scale (thermal assessment votes), we employed a cumulative mixed-effects model with a logit link and random intercepts, following the approach detailed by^66^. Our simulation-based power analysis indicated that, for medium to large effect sizes (i.e. 0.5 $\le$* Cohen’s d *$\le$* 0.8* , corresponding to odds ratios of approximately $1.5\le odds\, ratio \le 5$ according to^68^), the model achieved an average power of 82% across selected random intercept standard deviations ($\sigma = 1, 1.5, 2$) For large effect sizes, the power increased to 99% with 20 participates. For a detailed breakdown of the power analysis results, see Supplementary Materials, Section S.2.

### Experimental conditions

To minimize extraneous influences on participants’ prevailing circadian rhythms, throughout the study week participants were instructed to maintain a consistent sleep schedule, keep their usual (unrestricted) food intake, restrict stimulating beverages, refrain from vigorous exercise, and consume only water during exposure. The experiment was conducted from April to July 2024 in La Rochelle, with two participants tested per week. Figure 2b illustrates the experimental session timeline, including the light conditions, thermal conditions, and the sequence of programmed questionnaires. Each experimental session lasted approximately 3 h and 5 min. To ensure the integrity of the continuous data collection, participants were instructed to use the toilet before the session began; consequently, no breaks were permitted during the experiment. The research protocol was approved by the Ethics Committee at the University of Tours and Poitiers in France (Protocol No. CER-TP 2024-02-08) and adhered to the principles of the Declaration of Helsinki. Each participant was provided with written instructions and an information sheet and gave written informed consent to participate in the study.

#### Light

Before we discuss the lighting setup, we have to first define an essential metric when working with light in the circadian rhythm context, which is the *Equivalent Daylight Illuminance* (EDI). This quantity measures how effective a given light source is at affecting the body’s internal clock and non-visual responses^58^. It focuses on light’s ability to stimulate the *intrinsically photosensitive retinal ganglion cells* (ipRGCs), which are directly linked to the *suprachiasmatic nucleus* (SCN) the master of the internal clock^36^. EDI uses $S(\lambda )$, the ipRGCs spectral responsiveness, to quantify the proportion of the spectrum responsible for ipRGC activation, comparable to visual illuminance ($E^{visual}$) which relies on visual photoreceptors’ responsiveness $V(\lambda )$^69^. For the same light source when EDI is compared to visual illuminance it helps evaluate how closely an artificial light source approximates the spectral balance of daylight ($EDI_{daylight} \approx E^{visual}_{daylight}$ ), which offers an indirect measure of its potential to support non-visual biological responses, given the intensity. The selected bright light level is based on previous work by^70^, which summarized results from laboratory studies without the use of pupil dilators for long-term exposure (> 2 h). These results highlight that an EDI >250 lx, measured at eye level (1.2 m), is sufficient to elicit maximum melatonin suppression and achieve a phase shift of up to 3 h.

For this study, eight white standard illuminants (F5, F1, F7, D65, LED-B5, C, D75, D93)^71^ were emulated in terms of color appearance using four 60 $\times$ 60 cm LED panels (Hue Philips Surimu Panel). Achieving a target EDI > 250 lx at eye level inevitably leads to high horizontal illuminance. Therefore, the light condition offering the best balance between an adequate level of horizontal visual illumination and sufficient EDI at eye level was selected. In this case, emulated D93 was found to be the optimal choice.

**Figure 3:**
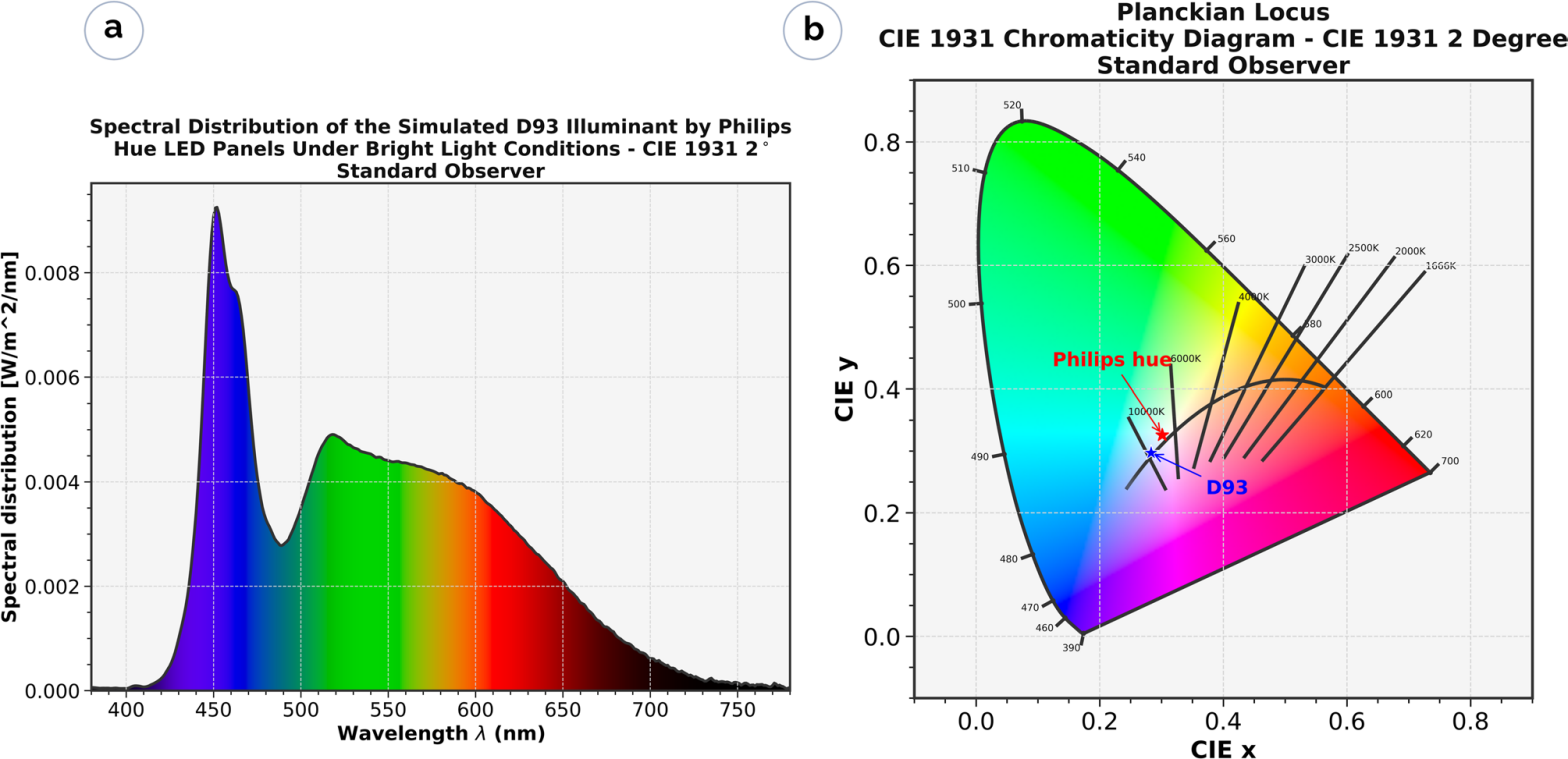
Spectral distribution of the emulated D93 light using LED panels (**a**) and its corresponding location on the chromaticity (x, y) coordinates (**b**), compared to the original D93 reference (Produced using^73^).

Figure 3a provides a visual summary of the obtained spectral distribution, and Fig. 3b shows the apparent color achieved by lighting system. The CIE chromaticity diagram in Fig. 3b indicate the position of the designed light relative to target illuminate. Spectral measurements were conducted in the windowless climate chamber designated for experimental sessions, utilizing PSR+ Spectroradiometer (accuracy = ±0.5 nm bandwidth, spectral resolution = 2.8 nm at 700 nm, range = 350 to 2500 nm) equipped with a right-angle diffuser (Field of View = ${180}^{\circ }$), positioned 1.2 m above the ground and 1.3 m vertically from the luminaires, aligned with the participant’s line of sight. The LED panels provided flexible control and enabled precise reproduction of lighting conditions through scripted commands via the Philips Hue API, allowing control over intensity and chromaticity. In this experiment, the original *x*, *y* chromaticity coordinates of D93 were input into the lighting system with the aim of closely replicating the apparent color of the target light. However, due to the spectral limitations of LED lights (e.g., the “cyan gap”) and the optical properties of the test room, we were not able to produce the exact apparent color of D93, but an approximate.

Table 2 provides a detailed summary of the calibrated lighting configuration, including correlated color temperature (CCT), vertical illuminance (lux), and equivalent daylight illuminance (EDI). All values were measured during the calibration phase and were held constant throughout the experimental sessions. While CCT is reported primarily for comparison purposes given its widespread use in previous studies it is not the most informative metric. The limitations of CCT as a descriptor of spectral characteristics will be further discussed in the limitation section. In the bright light session, the vertical EDI was set to 299 lx, and the horizontal illuminance at 848 lx, while in the dim light session, it was reduced to 39 lx and 111 lx respectively, to find a compromise between a relatively low EDI level to reduce the non visual light effects and sufficient lighting for regular desk tasks. Prior to any light (Dim or Bright) a dark adaptation session was conducted for this period, consisting of a vertical EDI of 5.57 lx. Dark adaptation is very important as it serves to normalize participants in terms of light pre-exposure as well as to sensitize their circadian system for greater response to the administrated light^62^. Participants completed digital questionnaires administered on a laptop. The screen display was set to night mode (yellowish) with brightness consistently set at 80% throughout all sessions.

**Table 2:** Correlated color temperature (CCT) and light parameters for bright, dim and dark adaptation. Light D93, chromaticity coordinates x = 0.28315, y = 0.297, CCT estimated using^72^ method.

Session	$CCT_{True}$ (K)	$CCT_{Estimated}$ (K)	Horizontal Illuminance (lx)	Horizontal EDI (lx)	Vertical EDI (lx)
Dark adaptation	9305	6848.01	15.45	16.09	5.57
Dim	–	7117.40	111.93	115.68	38.96
Bright	–	7200.70	848.22	891.93	299.42

#### Thermal environment

During the experiment, participants remained seated for the entire session. They completed questionnaires at scheduled intervals and engaged in quiet reading between questionnaires. For this sedentary activity, involving light desk work such as reading or typing, the metabolic rate was set to 1.1 met in accordance with ASHRAE Standard 55^74^. All participants were provided standardized summer clothing, corresponding to 0.3 clo, consisting of a sleeveless shirt (blue) and shorts (dark blue). Participants were informed about the overall procedure of the experimental session but were not made aware of the prevailing thermal conditions during the tests or the lighting changes (single-blinded study).

Each experimental session contained four distinct blocks see Fig. 2b :

**Block 0:** This 40-min period served as a combined thermal and dark adaptation phase, maintained at steady-state warm conditions (air temperature = $29.6 \pm 0.3 ^{\circ }C$) to reach a physiological steady state^75^. Concurrently, the very dim lighting (vertical EDI=5.57 lux) was implemented for a sufficient duration to allow for full retinal dark adaptation, a process shown to significantly increase circadian light sensitivity over a similar time-frame of 20–30 min^76^. Predicted mean vote (PMV) for both Fanger model^77^ and standard effective temperature (SET)^78^ based sensation model^79^ are $PMV_{Fanger} = 1.34 \pm 0.15$ and $PMV_{SET}= 0.80 \pm 0.09$ respectively. In this study, PMV is used to provide a reference characterization of the prevailing environmental conditions by integrating key variables, air temperature, mean radiant temperature, relative humidity, and air velocity into a single metric, rather than reflecting actual participants’ thermal sensation votes. After the adaptation period at the end of block 0, the light was switched on, and participants were continuously exposed to either bright or dim light for the remaining 2 h and 25 min of the experiment.

**Blocks 1–2–3:** Each of these three repeated blocks consisted of the same sequence of the following thermal conditions (see Fig. 2b):

- **Fan-at-constant-speed period:** Participants experienced 5 min of constant airflow from a fan (air velocity $= 0.2 \pm 0.1$ m/s) placed 1.10 m behind their chair with a set up similar to^80^. Figure 2c indicate the fan placement. They completed a thermal sensation, preference, pleasure, and comfort survey at the 1st, 3rd, and 5th min.
- **Skin-rewarming period:** A 10-min skin-rewarming period with the fan off allowing participants to return to their thermal state baseline. Same survey as fan-at-constant-speed period, completed at the 1st, 3rd, 5th and 8th min.
- **Fan-free-control period:** In this period, no surveys were administered; instead, participants were allowed to control the fan speed (air velocity $= 0.3 \pm 0.1$ m/s) via a wireless device (Fig. 2c) to achieve their personal comfort level. The current drawn by the fan served as an indicator of behavioral adjustment to warm conditions.
- **Steady-state period:** In blocks 1 and 2, this period was set to 30-min steady-state condition without fan operation. Participants completed thermal sensation, comfort, preference surveys at the 10th, 20th, and 25th min. In block 3, this period was shortened to 10 min, and the surveys was completed at the 9th min instead.

Table 3 gives a detailed break down of environmental conditions occurring in each block. The duration of each period was based on previous work^10^, indicating that 5 min of fan use is sufficient to alter skin temperature, while 10 min of rewarming allows values to return to baseline. The air temperature was controlled using radiant walls of AIRDIFF climate chamber at La Rochelle University ($3.28\times 2.69\times 2.5$m).

**Table 3:** Environmental variables with mean ± standard deviation for each period averaged across testing condition of light and time of the day.

Period	Time-steps (min)	${\textrm{T}_{\textrm{air}}}$ (${}^{\circ }$C)	${\textrm{T}_{\textrm{mrt}}}$ (${}^{\circ }$C)	${\textrm{v}}$ (m/s)	${\textrm{RH}}$ (%)	${\textrm{PMV}_{\textrm{fanger}}}$	${\textrm{PMV}_{\textrm{SET}}}$
Adaptation	0–39	29.6 ± 0.3	30.7 ± 0.4	0.0 ± 0.0	35.3 ± 8.5	1.3 ± 0.2	0.8 ± 0.1
Fan-at-constant-speed	40–45, 96–101, 152–157	29.9 ± 0.3	30.8 ± 0.5	0.2 ± 0.1	34.5 ± 8.4	1.1 ± 0.2	0.7 ± 0.1
Skin-rewarming	46–56, 102–112, 158–168	29.8 ± 0.3	30.8 ± 0.4	0.1 ± 0.0	34.6 ± 8.4	1.4 ± 0.2	0.8 ± 0.1
Fan-free-control	56–66, 113–123, 169–179	30.0 ± 0.3	30.8 ± 0.5	0.3 ± 0.1	34.4 ± 8.4	0.9 ± 0.2	0.7 ± 0.1
Steady-state	67–96, 123–152, 179–189	29.7 ± 0.3	30.9 ± 0.4	0.0 ± 0.0	34.3 ± 8.3	1.4 ± 0.2	0.8 ± 0.1

$T_{air}$, Air temperature; $T_{mrt}$, Mean radiant temperature; *v*, Air velocity (m/s); *RH*, Relative humidity; $PMV_{fanger}$, Predicted Mean Vote (Fanger model); $PMV_{SET}$, Predicted Mean Vote (SET model)

### Measurements

Figure 2c illustrates the experimental layout in detail. The participants were seated in the center of the climate chamber, just below a 4 $\times$ 4 grid of luminaries mounted on a ceiling of 2.5 m height, and surrounded by three instrumented columns. Two columns are positioned to the side to measure air temperature using type K thermocouples (chromel/alumel, d=0.8 mm, accuracy= ± 1.5 $\phantom{0}^\circ$C, range= -75$\phantom{0}^\circ$C to 250$\phantom{0}^\circ$C, time response ($t_{r}$) = 0.1 s) at four levels: 0.10 m, 0.60 m, 1.10 m, and 1.70 m^81^. The side columns also include additional sensors at 1.10 m height to measure relative humidity (HMP110 humidity and temperature probe, accuracy = ± 1.5% relative humidity, range=0 to 90%, $t_{r}$ = 17 s), globe temperature (type K, globe diameter=5 cm) and air velocity using a hot wire anemometer (air velocity transducers model 8475, accuracy = ±3.0% of the reading and ±1.0% of the full scale range=0.05 to 2.54 m/s, $t_{r}$ = 5 s). The third column placed 30 cm behind the chair is equipped with 4 hot wire anemometers, placed at 1.10, 0.60 and 0.10, with an additional sensor at 1.10 m, to measure the air velocity induced by the fan located behind. On the table, a supplementary globe thermometer with a larger diameter (d=21 cm) was added, along with a lux meter (HD2021T Transmitter, accuracy= ± 5 % , range=0.2 to 20 000 lx) to monitor illuminance during the sessions. All environmental measurements were sampled at a frequency of 1 min. To measure the current drawn by the fan, a low-resistance resistor (0.58 $\Omega$) was mounted in series with the fan. The voltage across the resistor was sent to a logger and then converted to current using Ohm’s law.

During each session, participants’ skin temperature was measured using two devices with a frequency of 1 min. Type T thermocouple (copper/constantan, d= 0.2 mm, accuracy= ± 0.5 $\phantom{0}^\circ$C, range=-40$\phantom{0}^\circ$C to +125$\phantom{0}^\circ$C, $t_{r}$ =0.01 s) placed on the hand, neck and scapula. Additionally, the BioNomadix$\textcircled {\hbox {R}}$ Skin Temperature Logger was used, with skin temperature sensors (accuracy=± 0.2 $\phantom{0}^\circ$C, 9.8 mm (diameter) $\times$ 3.3 mm (high), $t_{r}$ = 1.1 s ) placed on the chest, arm, thigh, and tibia (Fig. 2b). These sensors were fixed onto the skin with a breathable medical tape. Additional physiological measurements, including electrodermal activity, photoplethysmography, and blood flow via Doppler flowmetry, were also recorded. However, these will not be discussed in this work, as the primary focus will be on skin temperature. The presented results of environmental variables are obtained as follows: air temperature is averaged over eight sensors placed at aforementioned heights and positions; mean radiant temperature (MRT) is calculated as the average of the MRT values from small globes, computed using the formula implemented in^82^, and the MRT calculated from a large globe, using the formula from^81^; and air velocity is averaged using data from six anemometers, with four positioned behind and two to the sides of the participants. These averaged values are then used as inputs for the PMV model and the SET implemented in^82^. For mean skin temperature (MST) calculation during steady-state condition, we adopt the Ramanathan formula^83^, as it offers similar reliability to formulas that require 10–17 measurement points while using fewer measurements^84^.

1 $$\begin{aligned} MST_{Ramanathan} = 0.3 \cdot T_{Chest} + 0.3 \cdot T_{Arm} + 0.2 \cdot T_{Tibia} + 0.2 \cdot T_{Thigh} \end{aligned}$$

While for dynamic conditions the MST is calculated using ISO standards^85^, this formula incorporates skin regions directly exposed to fan flow (such as the neck and scapula), providing a more accurate representation of dynamic changes in skin temperature.

2 $$\begin{aligned} MST_{ISO} = 0.28 \cdot T_{Tibia} + 0.28 \cdot T_{Scapula} + 0.16 \cdot T_{Hand} + 0.28 \cdot T_{Neck} \end{aligned}$$

### Questionnaires

During the experimental regimes, two sets of questionnaires were administered to the participants. In dynamic conditions (Fan-at-constant-speed and Skin-rewarming, excluding Fan-free-control), participants responded to questions on thermal sensation, comfort, pleasure, and preference. In steady-state conditions, participants responded to the same set of questionnaires, except for thermal pleasure, as this phenomena is predominant in dynamic conditions alleviating discomfort under allesthesia^86^ (Table 4).

**Table 4:** English translations of the thermal subjective assessment scales^87^^80^.

Metric	Thermal sensation	Thermal comfort	Thermal pleasure	Thermal preference
Question	How are you feeling now?	Do you find this?	Do you consider this?	How would you prefer to be now?
Scale	Responses	–	–	–
− 3	Cold	Very uncomfortable	Very unpleasant	
− 2	Cool	Uncomfortable	Unpleasant	
− 1	Slightly cool	Slightly uncomfortable	Slightly unpleasant	Cooler
0	Neutral	Indifferent	Indifferent	No change
+ 1	Slightly warm	Slightly comfortable	Slightly pleasant	Warmer
+ 2	Warm	Comfortable	Pleasant	
+ 3	Hot	Very comfortable	Very pleasant	

In this study, they were administered in French see s.3/Supplementary Materials.

### Statistical analysis

All statistical analyses were performed in RStudio^88^ (R version 4.3.1). Cumulative mixed-effects models implemented in the ordinal package^89^, were used to analyze thermal assessment votes (sensation and comfort). In addition, continuous outcomes (mean skin temperature and the current drawn by the fan) were modeled using Gaussian linear mixed-effects models fitted with the lmerTest package^90^.

Model development involved two stages. First, four saturated model structures were compared.

- Model 1: fixed effects only.
- Model 2: included participant-specific random intercepts.
- Model 3: included both participant-level random intercepts and random slopes for the time-of-day, light and their interaction.
- Model 4: retained Model 3’s random effects structure and included a fixed three-way time-of-day $\times$ light-condition $\times$ experimental-block interaction.

The saturated model encompassed all candidate predictors of thermal assessment, skin temperature, and fan adjustment: exposure conditions (Light, Time-of-Day, and Light $\times$ Time-of-Day), thermal environmental indicator (PMV), anthropometric measures (BMI^91^ and BSA^92^), individual characteristics (sex and chronotype), and temporal factors explaining variance over time (day number for long-term effects and experimental block for short-term within-session evolution).

Secondly, the best-performing saturated model structure, identified using the Akaike Information Criterion (AIC)^93^ and supported by likelihood ratio tests, was selected for the next stage. A subsequent exhaustive AIC-based search (glmulti library^94^), was then performed to derive a reduced model that retained only essential predictors. The reduced model was adopted only if it provided a superior fit compared to the saturated model; otherwise, the saturated model was used.

Lastly, the best model identified underwent assumption violations checks. For Gaussian linear mixed-effects models, the performance library^95^ was used to assess linearity, homoscedasticity, influential observations, collinearity, and normality of residuals and random effects. For the ordinal regression models, goodness of fit was assessed by comparing the proportional odds model (cumulative logit link, which assumes equal predictor effects across response thresholds) to a multinomial logistic regression model (predictor effects are free to vary across response thresholds). If the multinomial model demonstrated significantly better fit, this would indicate violations of the proportional odds assumption. These procedures were performed for each model of interest in each experimental regime.

## Results

**Figure 4:**
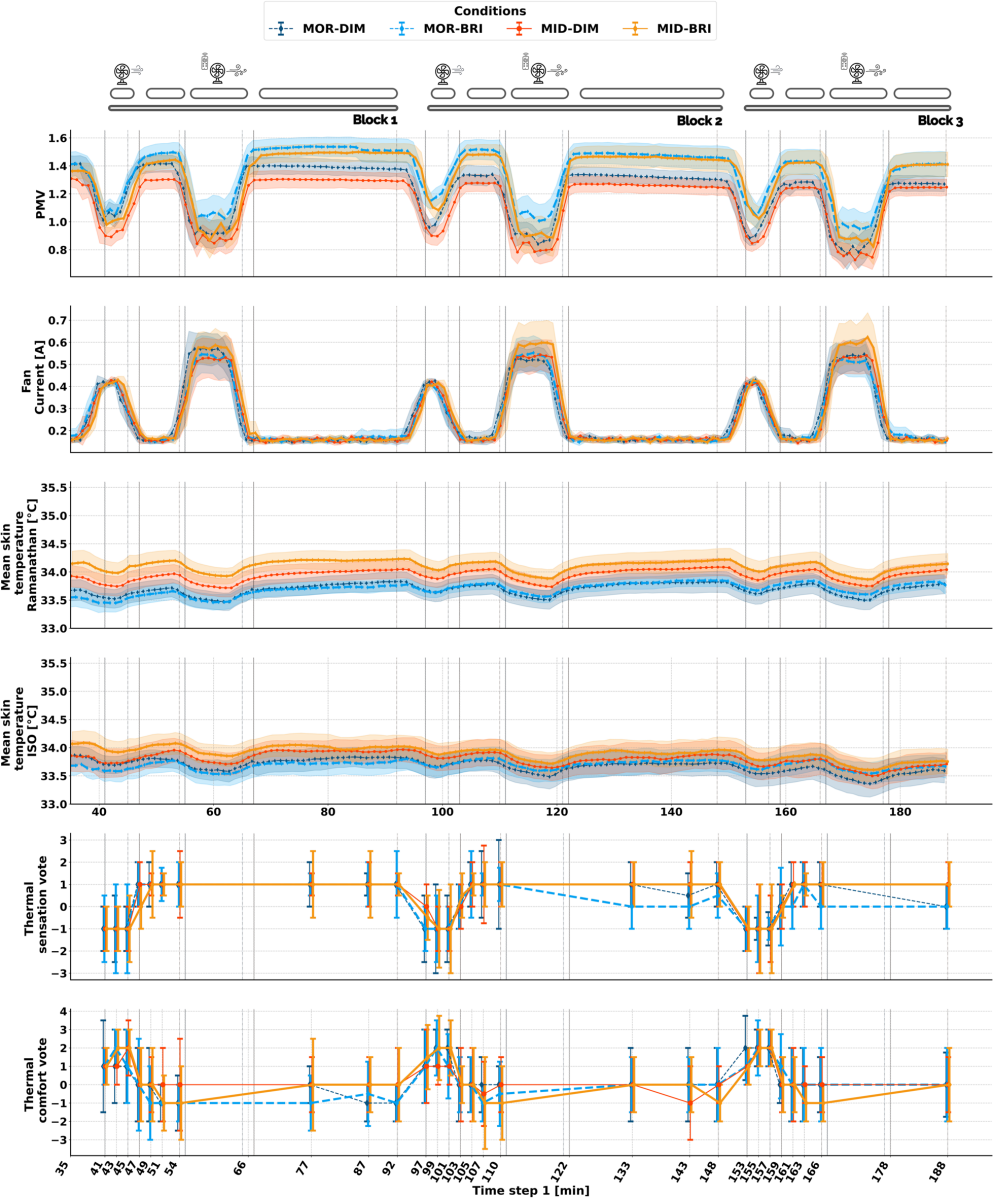
Time series of the observed PMV, fan current, and skin temperature (measured by Ramanathan and ISO methods), shown as means with 95% confidence intervals across all test conditions. Thermal sensation and thermal comfort votes are presented as medians with interquartile ranges. The small stripes mark the experimental regimes and blocks similar to Fig. 2b. The color coding of lines indicates the tested conditions of light and time of day.

Figure 4 summarizes the observed thermal conditions, fan adjustments, skin temperatures, and subjective thermal assessments recorded during the experimental sessions under different time-of-day and light conditions for 19 participants (data from one participant were excluded due to sickness).

Thermal assessment votes are represented in terms of median and interquartile ranges, as this is a more suitable way to summaries ordinal data^96^. The most dominant thermal sensation vote (TSV) was slightly warm (TSV = 1), primarily during steady-state and skin-rewarming regimes, with a corresponding thermal comfort vote ranging from indifferent to slightly uncomfortable ($0\le TCV \le -1$). During the fan-at-constant-speed period, the cooling effect of air movement induced a shift in TSV to a slightly cool sensation (TSV = -1), with a corresponding TCV ranging from slightly comfortable to comfortable ($1\le TCV \le 2$). During the periods in Blocks 2 and 3, there was a noticeable drop in thermal sensation votes for the morning sessions, especially under bright light, where the median TSV was 0 indicating a neutral vote. Comfort ratings varied among the experimental regimes, with the best comfort ratings observed during the constant fan-speed period, gradually declining during the skin-rewarming phase and stabilizing during the steady-state period. Pleasure ratings closely mirrored the trends observed in comfort (exhibiting increases during fan exposure and declines during the skin-rewarming phase). Preference ratings revealed that participant preferred to be cooler throughout the session, while they preferred no change during the fan intervention. Given the strong alignment between pleasure and comfort trends, as well as the conceptual redundancy of analyzing preference independently, pleasure and preference ratings were excluded from subsequent statistical analysis to streamline the focus on thermal sensation and comfort. The changes in thermal comfort induced by experimental exposure (light and time of day ) were not as visually distinct as those observed in thermal sensation. Accordingly, the following sections will present a detailed statistical analysis of these effects.

In accordance with thermal assessment votes, the PMV also highlights the different experimental regimes, showing a decrease during the fan-at-constant-speed period and a greater drop during free control both supported by the observed fan current patterns. Regarding fan adjustment, under the bright midday condition, fan speed was progressively increased as the session advanced.

As noted previously, PMV is higher in the bright sessions due to the radiant heat emitted by the luminaire panels, which significantly elevated the mean radiant temperature. Importantly, statistical models are designed to handle variability. By including PMV as a confounder, the model controls for these thermal influences ensuring the estimated effects of light and time of day are accurate and isolated.

The MST trend aligns with the experimental regimes: a drop in MST during the fan-at-constant-speed phase, followed by a return to baseline levels during rewarming. Subsequently, skin temperature drops during the free fan-control phase for an extended period before stabilizing in the steady-state phase. This pattern holds true for both the Ramanathan and ISO MST. After 40 min of adaptation, and given the prevailing thermal conditions we would expect MST to follow an identical pattern as observed in PMV with MST bright morning being the highest and dim midday to be lowest. Instead, the bright midday session had the highest observed MST levels among the test conditions, followed by the dim midday session, and then the morning sessions. Notably, the bright morning skin temperature was lower at the beginning of the sessions, even before the light was turned on, and this difference persisted even after 40 min of thermal adaptation, indicating that the effect extends beyond mere pre-exposure influences^97^ and supports the idea that it may be driven by circadian related variations in skin temperature set point^9^. For the ISO formula, skin temperature seems to converge at the end of the experiment among test conditions. Whereas for the Ramanathan formula, differences persist between morning and midday sessions. In the morning, despite the fact that bright light session is considered to be warmest on average as indicate by PMV, the MST Ramanathan remains at a level similar to the dim light condition, with a slight increase at the end of the session.

### Skin temperature

Three separate Gaussian linear mixed-effects models were fitted to examine mean skin temperature responses under three experimental regimes: steady-state conditions, fan-at-constant-speed, and skin-rewarming. For each regime, data points recorded between successive votes (including the vote events) were extracted according to their respective regime and used as input for the corresponding model. Each model included fixed effects for time of day (with morning as the reference), light exposure (with dim as the reference), experimental block (with the first block as the reference), and the Fanger PMV. In addition, the model for the fan-at-constant-speed condition included sex, and during the skin-rewarming period included BSA, based on their contribution to model fit as indicated by feature selection (Statistical analysis). In the steady-state model, MST Ramanathan is used as the outcome while in dynamic conditions model MST ISO is adopted instead. All models incorporated subject-specific random intercepts and slopes for the interaction of time of day and light to account for inter-individual variability in terms of response to light at different times of day.

**Figure 5:**
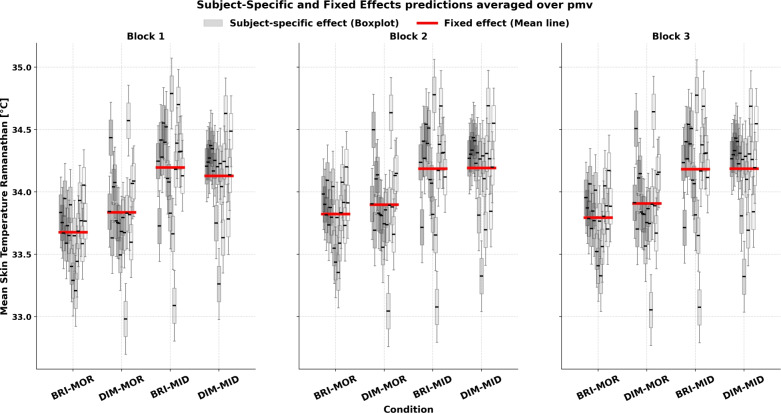
Subject-specific and average predictions of Ramanathan mean skin temperature under steady-state condition. Gray boxplots represent individual subject-specific predicted responses over range of observed pmv, while the red line represents the fixed-effects prediction averaged over pmv. Data are stratified by light exposure and time of day interaction, with separate panels for each experimental block.

Table 5 resumes the obtained predictors’ standardized estimates for the MST model across the tested regimes. The fixed effects for the midday session (relative to the morning baseline) are consistently positive and significant, indicating that skin temperature is higher at midday. In contrast, bright light (compared to dim light) shows a significant negative main effect on mean skin temperature. Furthermore, the experimental block effects (2 and 3) are positive and significant, suggesting that skin temperature increases as the session progresses. The Fanger PMV is also a strong positive predictor, contributing to increased skin temperature. Notably, the two-way interaction between midday and bright light is positive and significant. This indicates that although bright light alone tends to lower skin temperature, its effect is counterbalanced during midday so that there is no resulting cooling effect. While bright light have a significant cooling effect when administrated in the morning, over time (in later blocks) it still contributes to a net warming effect (significant positive two-way interaction Light$\times$Block). Summing up the categorical effects for each condition at different experimental blocks, we observe that: under steady-state conditions, the bright morning session shows lower skin temperatures compared to the baseline dim morning session across all blocks. Figure 5 presents a graphical representation of the model predictions in steady-state, illustrating both the fixed effects averaged over observed PMV range and subject-specific predictions. The figure shows that, despite higher PMV values (which the model controls for), the bright morning condition consistently predicts lower MST levels. In the fan-at-constant-speed regime, this effect is most evident in block 1, with only a slight increase seen in block 3, though the overall magnitude remains small. In the skin-rewarming regime, the bright morning condition exhibits the lowest skin temperature in blocks 1 and 2, whereas the bright midday condition consistently shows the highest temperature levels (compared to all sessions).

Additionally, the interaction terms between time of day and block are significant and negative, especially in the skin-rewarming period. The three-way interactions are also negative and significant, indicating that even though the bright midday session maintains the highest absolute temperature levels, the incremental effects of the experimental conditions diminish in later blocks. During the dynamic conditions, feature selection yielded additional predictors. In the fan-at-constant-speed model, sex was included, showing that males had lower skin temperatures (although this effect was not significant). In contrast, during the skin-rewarming condition, BSA had a significant negative effect on skin temperature, indicating that individuals with higher BSA experience lower mean skin temperatures during the rewarming period.

The random effects of the model reveal considerable variability across participants (see Supplementary Materials, Section S.4 for full model summary). Specifically, there is substantial variation in the intercept (baseline values), suggesting that different participants start at different baseline levels. The effects of light, time of day, and their interaction also vary between subjects, indicating that the impact of these factors on the outcome is not uniform across individuals. Figure 5 illustrates this between-subject variability, shown by the spread in the predicted subject-specific responses across the range of observed PMV values during each test condition. This individual variability was consistently observed across all models, as detailed in the following section. For more information on the random effects of the presented models, please refer to Supplementary Materials, Section S.4.

**Table 5:** Fixed effects estimates for mean skin temperature response models during steady-state , fan-at-constant-speed and rewarming period.

Parameter	Steady-state	Fan-at-constant-speed	Skin-rewarming
Intercept	$-0.41\,(0.20)$	$-0.11\,(0.24)$	$-0.43\,(0.17)^{*}$
Time of Day (MID)	$$0.74\,(0.17)^{***}$$	$$0.51\,(0.18)^{*}$$	$$0.83\,(0.17)^{***}$$
Light (BRI)	$$-0.41\,(0.17)^{*}$$	$$-0.35\,(0.11)^{**}$$	$$-0.39\,(0.12)^{**}$$
Block 2	$$0.16\,(0.02)^{***}$$	$$0.23\,(0.06)^{***}$$	$$0.26\,(0.04)^{***}$$
Block 3	$$0.18\,(0.06)^{**}$$	$$0.24\,(0.06)^{***}$$	$$0.40\,(0.05)^{***}$$
PMV	$$0.30\,(0.02)^{***}$$	$$0.13\,(0.02)^{***}$$	$$0.36\,(0.03)^{***}$$
sex (Male)	–	$-0.53\,(0.26)^{\cdot }$	–
BSA	–	–	$$-0.36\,(0.12)^{**}$$
MID $$\times$$ BRI	$$0.58\,(0.24)^{*}$$	$$0.90\,(0.27)^{**}$$	$$0.63\,(0.23)^{*}$$
MID $$\times$$ Block 2	$$0.00\,(0.03)$$	$$0.00\,(0.09)$$	$$-0.21\,(0.06)^{***}$$
MID $$\times$$ Block 3	$$-0.03\,(0.08)$$	$$-0.17\,(0.09)^{*}$$	$$-0.37\,(0.07)^{***}$$
BRI $$\times$$ Block 2	$$0.21\,(0.03)^{***}$$	$$0.14\,(0.09)$$	$$0.03\,(0.06)$$
BRI $$\times$$ Block 3	$$0.12\,(0.08)$$	$$0.30\,(0.09)^{***}$$	$$0.21\,(0.07)^{**}$$
MID $$\times$$ BRI $$\times$$ Block 2	$$-0.40\,(0.04)^{***}$$	$$-0.61\,(0.12)^{***}$$	$$-0.47\,(0.08)^{***}$$
MID $$\times$$ BRI $$\times$$ Block 3	$$-0.30\,(0.12)^{**}$$	$$-0.74\,(0.12)^{***}$$	$$-0.66\,(0.09)^{***}$$
Standard errors are in parentheses $$\beta (se(\beta ))$$. Significance codes: $$^{***}p<0.001$$, $$^{**}p<0.01$$, $$^{*}p<0.05$$, $$\cdot \,p<0.1$$.			

### Thermal sensation

**Figure 6:**
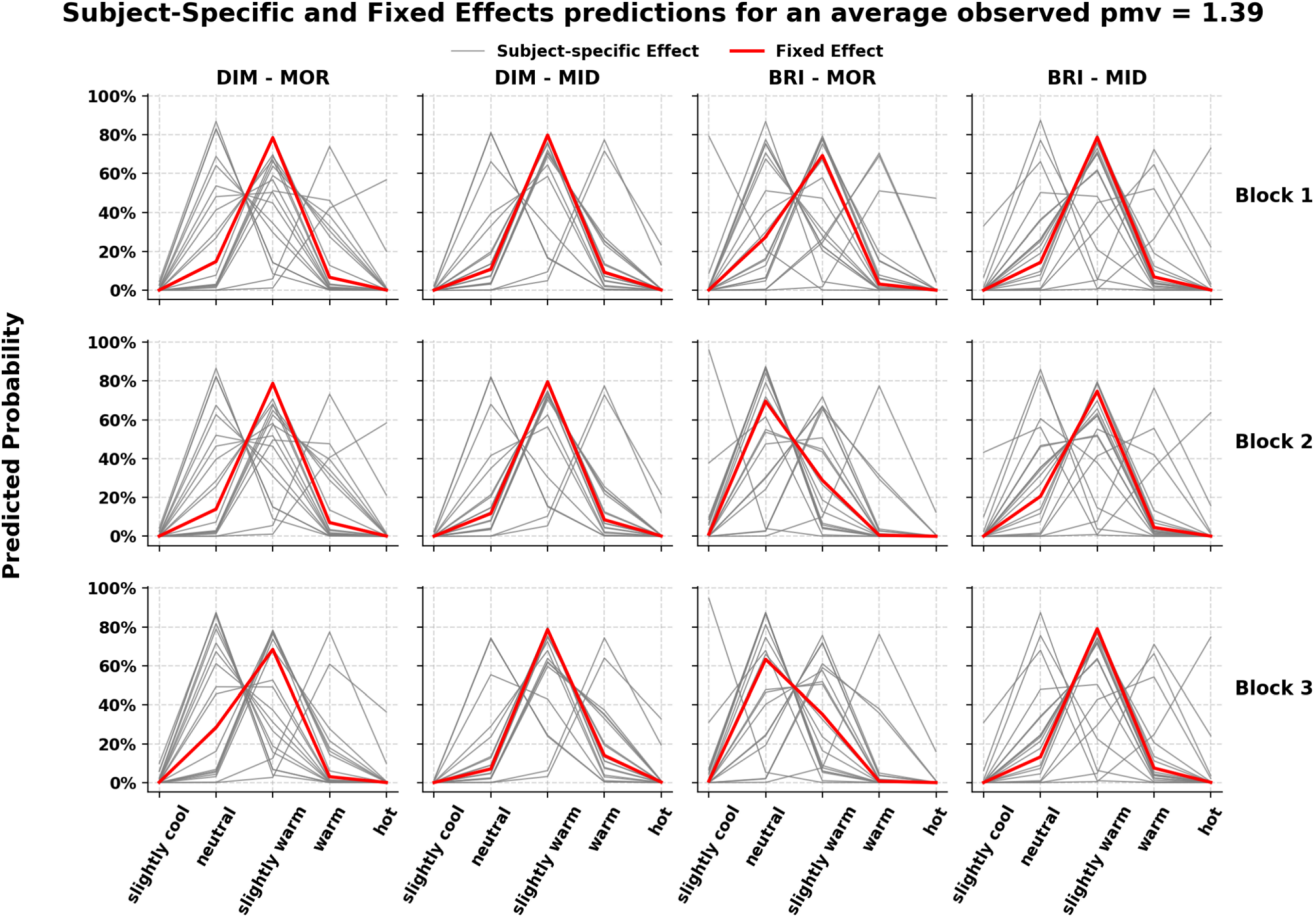
Subject-specific and average predictions of probability for thermal sensation categories under steady-state condition. Gray lines represent individual subject-specific predicted responses, while the red line represents the fixed-effects average prediction. Data are stratified by light exposure and time of day, with separate panels for each experimental block.

Table 6 summarizes the standardized log-odds coefficient for the ordinal thermal sensation models across the tested experimental regimes, with all models including subject-specific random intercepts and slopes for the time-of-day and light interaction. The main effect of bright light was non-significant in the steady-state and fan-at-constant speed regimes, though the directionality aligned with the hypothesis (cooling effect in the morning). Notably, in the skin-rewarming regime, bright light showed a significant cooling effect. Two- and three-way interaction terms were non-significant, except for the Light $\times$ Block 2 interaction in the steady-state regime, corresponding to an odds ratio of OR=0.15. This indicates a pronounced shift toward cooler thermal sensation votes (75% reduction in the odds of warmer TSV compared to dim morning at Block 1) during morning sessions in Block 2, with a weaker, non-significant trend in Block 3. Figure 6 presents the subject-specific model predictions (gray lines) and the fixed-effects model predictions (red line, excluding random effects) across different combinations of time of day, light exposure, and experimental blocks. The model predictions clearly show a substantial shift from slightly warm to neutral during bright mornings in Blocks 2 and 3, consistent with the model parameters and the observed trend in Fig. 4. The subject-specific effects also highlight the variability among individual responses, underscoring differences in thermal perception and responsiveness to bright light. Over experimental blocks, thermal sensation declined significantly in dynamic regimes, particularly in Block 3. An increase Fanger PMV value indicate a shift toward warmer votes reaching significance in steady-state and skin-rewarming, but not in the fan-at-constant-speed period.

Model comparisons for skin-rewarming favored the two-way interaction structure, with additional factors and covariates, factors like Day Number revealed gradual sensation decreases over days, with Day 4 showing a significant negative effect on sensation votes, suggesting a form of adaptation. Significant sex difference indicate that males felt cooler than females, higher BSA was associated with a significant shift toward warmer thermal sensation votes.

**Table 6:** Fixed effects estimates for ordinal thermal sensation models during steady-state, fan-at-constant-speed and rewarming period.

Parameter	Steady-state	Fan-at-constant-speed	Skin-rewarming
Time of Day (MID)	$0.36\,(0.61)$	$0.27\,(0.58)$	$-0.01\,(0.45)$
Light (BRI)	$-0.78\,(0.75)$	$-0.43\,(0.59)$	$-0.72\,(0.28)^{*}$
Block 2	$0.07\,(0.45)$	$-0.21\,(0.33)$	$-0.37\,(0.23)$
Block 3	$$-0.83\,(0.68)$$	$$-1.14\,(0.35)^{**}$$	$$-0.69\,(0.24)^{**}$$
Fanger PMV	$$1.06\,(0.38)^{**}$$	$$0.06\,(0.13)$$	$$0.80\,(0.15)^{***}$$
MID $\times$ BRI	$0.45\,(0.81)$	$0.15\,(0.66)$	$0.61\,(0.38)$
MID $\times$ Block 2	$-0.16\,(0.60)$	$-0.13\,(0.46)$	$0.21\,(0.33)$
MID $\times$ Block 3	$1.29\,(0.89)$	$0.07\,(0.47)$	$0.42\,(0.33)$
BRI $$\times$$ Block 2	$$-1.91\,(0.62)^{**}$$	–	–
BRI $\times$ Block 3	$-0.72\,(0.89)$	–	–
MID $\times$ BRI $\times$ Block 2	$1.57\,(0.86)^{\cdot }$	–	–
MID $\times$ BRI $\times$ Block 3	$0.35\,(1.23)$	–	–
Day 2	–	–	$-0.43\,(0.38)$
Day 3	–	–	$-0.45\,(0.30)$
Day 4	–	–	$-0.86\,(0.35)^{*}$
sex (Male)	–	–	$-2.12\,(0.94)^{*}$
BSA	–	–	$$1.51\,(0.54)^{**}$$

Threshold coefficients			
	Steady-state	Fan-at-constant-speed	Skin-rewarming
Thresholds	Slightly cool|neutral: $$-7.13\,(0.93)$$ Neutral|slightly warm: $$-1.73\,(0.78)$$ Slightly warm|warm: $$2.64\,(0.79)$$ Warm|hot: $$6.77\,(0.91)$$	Cool|slightly cool: $$-3.80\,(1.13)^{***}$$ Slightly cool|neutral: $$1.35\,(1.12)$$ Neutral|slightly warm: $$5.68\,(1.14)^{***}$$	Cool|slightly cool: $$-8.11\,(0.87)^{***}$$ Slightly cool|neutral: $$-6.19\,(0.82)^{***}$$ Neutral|slightly warm: $$-3.11\,(0.80)^{***}$$ Slightly warm|warm: $$0.24\,(0.79)$$ Warm|hot: $$3.51\,(0.82)^{***}$$

Standard errors are in parentheses $$\beta (se(\beta ))$$ . Significance codes:$$^{***}p<0.001$$, $$^{**}p<0.01$$, $$^{*}p<0.05$$, $$^{\cdot }p<0.1$$

### Thermal comfort

Table 7 presents standardized log-odds ratio coefficients for our ordinal thermal comfort models across experimental regimes (steady-state, fan-at-constant speed, and skin-rewarming). These models retained the same subject-specific random effects structure as our thermal sensation models. However, model fit assessment to its multinational counter part indicated a possible violation of the proportional odds (PO) assumption, specifically the PO was partially violated for PMV in the steady-state and fan regimes and for experimental blocks in all regimes, with coefficients differing significantly across response categories. Despite these violations, all remaining predictors, including time of day, light interactions, and individual factors (BSA, BMI), showed consistent directional effects across categories, as confirmed by overlapping posterior credible intervals in our bayesian sensitivity analyses (see Supplementary Materials, Section S.5 for details). These analyses decomposed the ordinal response into a series of binary logistic models, comparing posterior distributions of coefficients across adjacent categories.

While the PO violations warrant caution for category-specific interpretations of PMV and block effects, the ordinal models remain valid for aggregate inferences due to robustness checks and the directional stability of the remaining predictors. For the comfort model, time of day exhibits a positive but non-significant effect, the fixed effect of light is positive and significant in the steady-state and skin-rewarming models, indicating a shift toward higher comfort vote categories, while no significant effect of light is observed in the fan-at-constant-speed model, which aligns with our thermal sensation findings (i.e., cooler sensation votes yield higher comfort categories). The interaction of light and time of day is non-significant but trends similarly to the sensation models, suggesting that bright light predominantly influences thermal comfort in the morning, with a counterbalancing effect at midday.

Although the PMV effect is category dependent, from a contextual standpoint, its directional impact in the steady-state and skin-rewarming models is significant and consistent with our sensation results: an increase in PMV is expected to yield lower comfort (i.e., higher discomfort) vote categories, while the magnitude is small and non-significant in the fan-at-constant-speed model. The block effects similarly indicate that comfort improves in later blocks compared to the first, mirroring the trend of lower sensation votes over time. However, the two-way interaction estimates for blocks are inconsistent with the sensation models and likely reflect category-specific effects. Thus, we refrain from making definitive claims regarding the temporal effects of experimental blocks.

Finally, in the fan-at-constant-speed model, feature selection yielded additional predictors. In this model, BSA exhibits a significant negative effect, while BMI shows a significant positive effect. Notably, the feature selection process appears to have eliminated sex as a confounder, since the effects for BMI and BSA remained consistent regardless of its inclusion. In the skin-rewarming model, convergence issues necessitated adjustments to the initial saturated model, replacing BSA and BMI with their originating covariates, weight and height. The revised model again indicates that adaptation occurs during skin-rewarming, with a significant shift toward higher comfort votes on the last day, in line with our sensation model findings. Additionally, weight had a significant negative effect, which corresponds with our sensation model that indicated warmer sensation votes for higher BSA.

**Table 7:** Fixed effects estimates for ordinal thermal comfort models during steady-state , fan-at-constant-speed and rewarming period.

Parameter	Steady-state	Fan-at-constant-speed	Skin-rewarming
Time of day (MID)	$0.84\,(0.80)$	$0.78\,(0.67)$	$0.06\,(0.51)$
Light (BRI)	$1.33\,(0.66)^{*}$	$0.88\,(0.73)$	$1.03\,(0.45)^{*}$
Block 2	$$0.89\,(0.29)^{**}$$	$$1.00\,(0.38)^{**}$$	$$0.25\,(0.16)$$
Block 3	$$0.91\,(0.43)^{*}$$	$$1.75\,(0.40)^{***}$$	$$0.46\,(0.17)^{**}$$
Fanger PMV	$$-0.78\,(0.32)^{*}$$	$$0.14\,(0.12)$$	$$-0.75\,(0.15)^{***}$$
MID $\times$ BRI	$-1.40\,(1.05)$	$0.21\,(0.94)$	$-0.95\,(0.69)$
MID $$\times$$ Block 2	$$-1.13\,(0.39)^{**}$$	$$-0.72\,(0.43)^{\cdot }$$	–
MID $\times$ Block 3	$-0.18\,(0.55)$	$-1.06\,(0.43)^{*}$	–
BRI $\times$ Block 2	–	$-1.09\,(0.43)^{*}$	–
BRI $\times$ Block 3	–	$-1.11\,(0.44)^{*}$	–
Day 2	–	–	$0.60\,(0.33)^{\cdot }$
Day 3	–	–	$0.74\,(0.39)^{\cdot }$
Day 4	–	–	$0.97\,(0.43)^{*}$
Weight	–	–	$-1.14\,(0.45)^{*}$
BSA	–	$$-2.37\,(0.39)^{***}$$	–
BMI	–	$$2.00\,(0.34)^{***}$$	–

Threshold coefficients			
	Steady-state	Fan-at-constant-speed	Skin-rewarming
Thresholds	Uncomfortable|Slightly Uncomfortable: $$-3.39\,(0.73)$$ Slightly uncomfortable|Indifferent: $$0.02\,(0.70)$$ Indifferent|Slightly comfortable: $$2.68\,(0.71)$$ Slightly comfortable|Comfortable: $$5.43\,(0.76)$$ Comfortable|Very comfortable: $$8.42\,(0.90)$$	Cool|Slightly cool: $$-3.80\,(1.13)^{***}$$ Slightly cool|Neutral: $$1.35\,(1.12)$$ Neutral|Slightly Warm: $$5.68\,(1.14)^{***}$$	Uncomfortable|Slightly uncomfortable: $$-2.78\,(0.59)^{***}$$ Slightly uncomfortable|Indifferent: $$0.21\,(0.59)$$ Indifferent|Slightly comfortable: $$2.32\,(0.59)^{***}$$ Slightly comfortable|Comfortable: $$4.92\,(0.62)^{***}$$ Comfortable|Very comfortable: $$8.34\,(0.74)^{***}$$

Standard errors are in parentheses $$\beta \,(se(\beta ))$$. Significance codes: $$^{***}p<0.001$$, $$^{**}p<0.01$$, $$^{*}p<0.05$$, $$^{\cdot }p<0.1$$

### Fan control

Table 8 summarizes the standardized fixed effects coefficients for the fan-control model, where fan current is used as the predicted outcome, retaining the same subject-specific random effects structure as in previous models. Data from 18 participants were included, with one participant excluded due to fan-control levels being identified as an outlier. In this model, feature selection did not improve the fit. Instead, the saturated model, which included three-way interactions, yielded the best fit based on AIC and differed significantly from the feature-selected model, as confirmed by a likelihood ratio test.

The main effects of light, time of day, and their interaction are small and non-significant, but their directionality aligns with the initial hypothesis. The effect of the experimental condition is significant, particularly in Block 2, where fan speeds are adjusted to lower levels compared to Block 1. Additionally, the two-way interaction between light and experimental block reveals a small but significant increase in fan adjustment during bright sessions in Block 2. In the midday session, there is an additional moderate increase in fan adjustment in Block 2, as indicated by the significant two-way interaction between time of day and Block 2. However, this effect is significantly attenuated during bright sessions as indicated by three way interaction. Air temperature was used in place of PMV, as PMV is partly a consequence of fan adjustment, which could lead to controversial interpretations. The model indicates that air temperature has a significant but small positive effect on fan speed, likely due to the controlled environment. Males tend to adjust the fan to higher speeds compared to females, with a significantly large effect. Chronotype also emerged as an influential factor; with equal proportions of participants in each chronotype category (Normal< Slight Late< Moderate Late, 6 participants per category), the results indicate that individuals with a Slight Late chronotype tend to adjust the fan to higher speeds, showing a significant and large effect. Individual characteristics such as BSA and BMI were not significant. A form of adaptation over days was identified as influential, with a reduction in fan speed observed relative to Day 1. Nevertheless, this effect was small and not statistically significant.

**Table 8:** Fixed effects estimates for the fan current response model during free control period.

Parameter	Estimate (SE)	Parameter	Estimate (SE)
Intercept	$-0.50\,(0.30)$	BSA	$-0.28\,(0.21)$
Time of day (MID)	$-0.12\,(0.15)$	BMI	$-0.03\,(0.15)$
Light (BRI)	$-0.24\,(0.13)^{\cdot }$	MID $\times$ BRI	$0.37\,(0.23)$
Block 2	$$-0.21\,(0.05)^{***}$$	MID $$\times$$ Block 2	$$0.31\,(0.08)^{***}$$
Block 3	$-0.04\,(0.06)$	MID $\times$ Block 3	$0.13\,(0.08)^{\cdot }$
Air temperature	$$0.16\,(0.03)^{***}$$	BRI$$\times$$ Block 2	$$0.25\,(0.08)^{**}$$
sex (Male)	$0.75\,(0.32)^{*}$	BRI$\times$ Block 3	$0.00\,(0.08)$
Chronotype (moderate late)	$-0.12\,(0.28)$	MID $\times$ BRI$\times$ Block 2	$-0.26\,(0.11)^{*}$
Chronotype (Slight late)	$$1.01\,(0.30)^{**}$$	MID $$\times$$ BRI $$\times$$ Block 3	$$0.12\,(0.11)$$
Day 2	$-0.17\,(0.09)^{\cdot }$		
Day 3	$-0.02\,(0.12)$		
Day 4	$-0.20\,(0.11)^{\cdot }$		

Standard errors are in parentheses $$\beta \,(se(\beta ))$$. Significance codes: $$^{***}p<0.001$$, $$^{**}p<0.01$$, $$^{*}p<0.05$$, $$^{\cdot }p<0.1$$

## Discussion

###  Skin temperature

The observed difference in MST between morning and midday primarily reflects circadian rhythmicity: both proximal and distal skin temperatures decline in the morning^19^ to reduce heat loss via peripheral vasoconstriction and promote the rise in core body temperature^17,20,98^. In the bright morning session, MST is the lowest among the test conditions, even before the light is switched on (see Fig. 4, t < 40 min). Despite the session being the warmest based on PMV, skin temperature remains at the same level as observed during the dim morning session. This suggests that bright light exposure suppresses the expected increase in skin temperature. This aligns with Harmsen et al.^99^ who observed bright-light-induced reductions in distal skin temperature, and Lok et al.^100^ who showed that a bright light environment during scheduled wake periods in a forced-desynchrony setting acutely reduces peripheral skin temperature, indicating a less vigorous engagement of vasodilation according to the *heat-gain/heat-loss* modes hypothesis^101^. This hypothesis, well-established in exercise science, suggests that during the morning heat-gain phase, when the circadian rhythm is actively elevating core body temperature, any additional warming from spontaneous activity or exercise elicits a weaker thermoregulatory cooling response^101^. The same circadian mechanism may also influence responses to passive (i.e. in sedentary contexts) heat exposures. Supporting this, Shoemaker and Refinetti^102^ found that male participants preferred higher ambient temperatures when their core temperatures were lower in the morning. Further evidence is provided in Fig. 1, which shows that the minimum in distal skin temperature coincides with the maximum rate of increase in core body temperature, underscoring the presence of this heat-gain mode.

### Thermal sensation and comfort

Our findings indicate that the first hypothesis is confirmed for both the steady-state and skin-rewarming periods. Although the main effects of light in these regimes are comparable in magnitude, significance is mainly observed in skin-rewarming (OR = 0.48, p < 0.05, 52% lower odds of being in the higher vote category compared to the baseline). In the steady-state condition, the effect of light is time-dependent and emerges later in the experiment (Light $\times$ Block 2, OR = 0.15, p < 0.001), with a cooling negative effect evident only in the morning, as all terms associated with the midday condition show positive effects in the sensation model (causing a shift toward warmer votes). Thermal comfort results further support these findings, with bright light exposure inducing a shift toward higher comfort votes in both the steady-state (OR = 3.78, p < 0.05, 3.78 times higher odds of being in a higher vote category compared to the baseline) and skin-rewarming (OR = 2.80, p < 0.05) periods. In contrast, under the fan-at-constant-speed regime, both thermal sensation and comfort indicate that the main effects of light, time of day, and their interaction are not significant, suggesting that the observed effects of light on thermal assessment are absent in slightly cool conditions.

The observed difference in thermal sensation goes in the same direction as prior research suggesting that cooler correlated color (i.e. bluish) temperatures are often associated with cooler thermal sensations^41,46,52,103^ compared to their warm counterpart (i.e. orangish), a pattern also noted in the meta-analysis by^104^. While these findings are part of studies conducted under the hue-heat hypothesis, cool CCTs (high blue content) are equivalent to a ’bright’ condition as they generally exhibit greater melanopic potential compared to warm CCTs, more akin to a ’dim’ condition, if intensity is held constant. In this regard, the observed patterns in previous works could be a circadian masking effect manifesting in the morning under warm conditions. Hence, the hue-heat hypothesis can also be regarded as a special case of the more comprehensive illuminance-thermal markers hypothesis.

While our results are in line with previous works, our findings cannot conclusively establish causality compared to prior studies. A key limitation lies in the reliance on CCT as a metric for characterizing light. CCT is not the best for this purpose, being a unidimensional quantity that assigns a color temperature to an artificial light^105^, it essentially arises from an optimization problem aimed at finding the nearest Planckian radiator in terms of color on the chromaticity diagram^106^. While color temperature is meaningful for Planckian radiators, it can be misleading for artificial lights far from the planckian locus, it results that two lights may share the same CCT despite differing in spectral distribution. New studies should adopt unified metrics such as EDI, as these are more directly tied to physiological responses, whereas color appearance may be confounded by interindividual variability in psychological color-associations^53,57^. Time of day must also be attributed great attention as the effect of light is heavily time-of-day dependent. Future research on light and thermal perception and evaluation should shift focus from the apparent color of light and instead prioritize how and when light affects human physiological responses.

Our findings do not fully support the second hypothesis. The interaction between time of day and light condition was consistently non-significant across models of thermal sensation and comfort. The warming effect of bright light is superimposed on the elevated MST present at midday due to circadian rhythm. This combined effect complicates efforts to disentangle non-visual influences of light on thermal perception, limiting assessment of light’s independent role despite controlling for the thermal environment. When skin temperature was treated as an output in MST models, the significant interaction term exhibited a sign opposite to that of the bright light condition across all models, systematically counteracting its effect, supporting the absence of a cooling effect during midday. Thus, while the results for MST are clear, the data remain inconclusive for rejecting the null hypothesis regarding the direct effect of light on thermal sensation during midday.

### Thermal behavior

The fan-control results are primarily influenced by factors such as sex, chronotype, and experimental blocks, with no significant effects associated with light, time of day, or their interaction. However, this does not definitively state that the effects of light, time of day, or their interactions are absent. According to our power analysis, the study was sufficiently powered to detect large effect sizes on continuous outcomes. The lack of significance suggests that any effects of light and time of day on fan use were likely subtle and minimal, and were overridden by the more impactful individual differences in sex and chronotype. The significant impact of sex is not surprising given that sex-linked differences in airflow sensitivity have been previously observed, for example in^107^. Although research on chronotype differences in thermal assessment is limited, previous studies demonstrated chronotype-based differences in heat-induced pain tolerance, with morning types exhibiting higher tolerance than evening types, regardless of time of day^108^. Our finding of chronotype influencing only fan control behavior does not rule out the existence of chronotype differences in thermal assessment; rather, any effect may be smaller or potentially undetected in our models due to limited sample sizes within chronotype categories. However, within the fan control model, the observed chronotype effect was evident and sufficiently distinct among subgroups. Notably, fan control behaviour showed a pattern suggestive of a similar trend to the pain tolerance findings: normal chronotypes tended to select lower fan speeds compared to slightly late chronotypes. The emergence of chronotype as an influential factor in the fan adjustment model warrants further attention, as chronotypes reflect a consistent state of individual entrainment that might affect overall decision^109^. Further research is needed, since variations in chronotype are linked to differences in the timing of thermoregulatory reactions throughout the circadian cycle.

### Individual variability

In this work, all inferential models improved in fit when including the interaction terms of light and time of day as random slopes, highlighting that individuals may respond differently to light and time of day, with some participants being more sensitive than others. For example, Phillips et al.^110^ exposed participants to increasing light levels and measured the point at which their melatonin production was reduced by 50 per cent. They found that some individuals needed over fifty times more light than others to reach this threshold, clearly showing that treating light sensitivity as a single constant would hide these enormous personal differences^110^. In terms of time of day, long-term monitoring of core body temperature in everyday settings has revealed that the clock time at which each person’s temperature drops to its daily lowest point can differ by two or more hours, and the size of the temperature swing over 24 h also varies greatly. These individual patterns are closely linked to chronotypes, with evening-types and morning-types each displaying distinct characteristic profiles of temperature change throughout the day^111^. While these examples highlight key physiological sources of variability in circadian light responses, they are not exhaustive. Other factors, such as psychological profiles, hormonal status, cultural background, may also contribute significantly to individual differences and present important opportunities for future research.

## Limitation

The primary limitation of this study and prior research investigating the effects of light on subjective thermal responses is the inherent challenge of disentangling the warming effect of lighting systems from their resulting circadian impact. Observing these effects requires high light intensities, which inevitably generate radiant heat from electrical components. In this study, LEDs were selected due to their low heat emission, and additional aluminum duct tape was applied to the luminaire frames to mitigate this issue; however, the mean radiant temperature remained partially influenced. Supplementary Materials, Section S.6 report the measured luminaire-frame temperatures. A clear distinction between the dim and bright sessions confirms the presences of greater radiant heat loads during the bright sessions. For future research, unless radiant heat gains are fully decoupled from light intensity, it will remain challenging to isolate and fully characterize the non-visual effects of light on thermal assessment, particularly during critical periods such as midday when MST is higher and light-driven thermal effects may be conflated with ambient conditions.

A second limitation is linked to statistical power. While the main effect of light in skin-rewarming period and its interaction with the experimental block in steady-state, were statistically significant, the study’s sample size provided sufficient power to detect only medium-to-large effect sizes for thermal assessment outcomes (see Supplementary Materials, Section S.2). However, smaller effects, such as the interaction between time of day, could not be reliably distinguished by the ordinal regression models. These subtler effects were likely masked by confounding radiant heat from the luminaires themselves.

We used artificial lighting in a windowless climate chamber to maximise experimental control and isolate mechanistic effects, this methodological choice is not a recommendation to substitute natural daylight, where feasible, exposure to daylight should be prioritized. Daylight is the principal driver of the physiological and perceptual responses observed in real-world settings, and previous work has highlighted the interplay between daylight and thermal perception^112,113^. In real environments artificial and natural light typically coexist. Accordingly, future work should replicate this protocol under natural daylight or with calibrated daylight-mimicking systems to establish external validity and to evaluate the influence of daylight on thermal perception.

Our aim was to determine the primary contribution of light and time of day to thermoregulatory changes under warm ambient conditions; therefore, the results are context-specific and may not generalize across seasons. Future studies should examine longer exposure durations (full day) and replicate this protocol, ideally informed by core-body temperature rhythms across different seasons^114^ to clarify seasonal dependency and the practical implications for heating and cooling demand.

Finally, The cohort’s homogeneity restricted to young healthy adults was a deliberate methodological choice. This design strengthens the internal robustness of the findings by reducing variance attributable to age-related or health-related confounders. However, we acknowledge that this same homogeneity limits the external validity and generalizability of our conclusions. Therefore, while this approach provides a clear effects for this specific demographic, extending these findings to a broader population requires future studies with larger, more diverse cohorts.

## Conclusion

This study investigated the effect of bright and dim light exposure on thermal assessment, skin temperature and behavioral adjustments (through fan control) at two times of the day, morning and midday under warm steady-state and dynamic conditions induced by a fan-at-constant-speed and skin-rewarming (after fan use). The major findings of this work highlight:

- The circadian nature of skin temperature, with mean skin temperatures lower in the early morning compared to midday, regardless of the prevailing regime, which explains the cooler sensation observed in the morning compared to midday.
- the effect of bright light exposure (vertical EDI $\approx$ 300 lux) in the morning inducing a sensation of coolness, with a shift toward neutral votes in a slightly warm environment after 2 h of exposure in steady-state conditions, and resulted in cooler sensation votes during the skin-rewarming period. Consistently, bright light resulted in improved thermal comfort votes in the morning compared to dim morning base line and midday for both regimes.
- The cooling effect of bright light in the morning being masked during controlled dynamic slightly cool conditions induced by the fan, where absence of this effect was observed consistently on both sensation and comfort.
- The main effect of bright light on behavioral adjustment (fan control) being marginally significant. However other factors, such as individual and temporal characteristics including sex and chronotype, and the warming effect over time, were more significant.

The current results indicate the potential of utilizing the circadian component of thermoregulation, emphasized by light exposure, to alter thermal sensation. However, these findings require further confirmation and a better understanding of the intra-individual variability in response to light exposure at different times of the day and of the temporal components of these responses. Within this circadian framework, adaptive control strategies that consider dual set points for light and temperature might become an option to create indoor environments that ensure comfort and align with human physiology.

## Supplementary Information

Supplementary Information.

## Data Availability

All raw and processed data generated in this study, including the primary datasets reported in the paper and the additional supporting measurements, will be made fully accessible. Upon publication, these data will be deposited in a public repository and described in a forthcoming Scientific Data paper. Once available, they can be accessed at 10.6084/m9.figshare.30244300.v1. In the meantime, all data supporting the results and conclusions presented here are available from the corresponding author upon reasonable request.
