# Supplementary material for: Acute effect of light and time of day on thermal physiology, perception, and behavior
Source: Sci Rep. 2025 Nov 4;15:38640. doi: 10.1038/s41598-025-22542-w (PMC12586677; doi:10.1038/s41598-025-22542-w)
Supplement: Supplementary file 1 — Supplementary Information. [file 41598_2025_22542_MOESM1_ESM.pdf]

Supplementary Materials

s.1

Supplementary Table 1: Review of studies on the effect of light intensity and color on thermal sensation (TSV), thermal comfort (TCV), and physiological responses (Phy).

Study	Sample Characteristics	Light Conditions	Enviromental Conditions	Observed effects
% Gu et al 2023	N = 20<br>$Female_{proportion}$ = 0.50<br>Age = 23.4<br>BMI = 21.8 Kg/m^2	CCT_{Cool} = NR K<br>CCT_{Warm} = NR K<br>CCT_{Fixed} = 6000.0 K<br>$Light_{Bright}$ = 950.0 lux<br>$Light_{Dim}$ = 550.0 lux<br>$Light_{Fixed}$ = NR lux	T_{Cool} = 22.0°C<br>T_{Warm} = 28.0 °C<br>T_{Fixed} = NR °C<br>RH = 55.35%<br>v_{air} = 0.1 m/s<br>State = steady<br>Clo = 0.5	Effect on TSV = ✗<br>Effect TCV : ✓<br>Effect Phy = ✗
Pigliautile et al 2023	N = 24<br>$Female_{proportion}$ = 0.42<br>Age = 23.5<br>BMI = NR Kg/m^2	CCT_{Cool} = 17800.0 K<br>CCT_{Warm} = 2010.0 K<br>CCT_{Fixed} = NR K<br>$Light_{Bright}$ = NR lux<br>$Light_{Dim}$ = NR lux<br>$Light_{Fixed}$ = NR lux	T_{Cool} = 18.0°C<br>T_{Warm} = 30.0 °C<br>T_{Fixed} = NR °C<br>RH = NR%<br>v_{air} = NR m/s<br>State = steady<br>Clo = NR	Effect on TSV = ✓<br>Effect TCV : ✓<br>Effect Phy = ✗
Baehr et al 2023	N = 12<br>$Female_{proportion}$ = 0.50<br>Age = 26.33<br>BMI = 23.01 Kg/m^2	CCT_{Cool} = NR K<br>CCT_{Warm} = NR K<br>CCT_{Fixed} = NR K<br>$Light_{Bright}$ = NR lux<br>$Light_{Dim}$ = NR lux<br>$Light_{Fixed}$ = NR lux	T_{Cool} = 24.5°C<br>T_{Warm} = 26.0 °C<br>T_{Fixed} = NR °C<br>RH = 27.5%<br>v_{air} = 0.1 m/s<br>State = steady<br>Clo = 0.75	Effect on TSV = ✓<br>Effect TCV : ✓<br>Effect Phy = ✗
Luo et al 2023	N = 16<br>$Female_{proportion}$ = 0.50<br>Age = 23.7<br>BMI = 21.9 Kg/m^2	CCT_{Cool} = 5700.0 K<br>CCT_{Warm} = 2700.0 K<br>CCT_{Fixed} = NR K<br>$Light_{Bright}$ = NR lux<br>$Light_{Dim}$ = NR lux<br>$Light_{Fixed}$ = 500.0 lux	T_{Cool} = NR°C<br>T_{Warm} = NR °C<br>T_{Fixed} = 17.0 °C<br>RH = 44.0%<br>v_{air} = 0.2 m/s<br>State = steady<br>Clo = 0.8	Effect on TSV = ✓<br>Effect TCV : ✓<br>Effect Phy = ✗

Supplementary Table 1: Review of studies on the effect of light intensity and color on thermal sensation (TSV), thermal comfort (TCV), and physiological responses (Phy).

Study	Sample Characteristics	Light Conditions	Enviromental Conditions	Observed effects
Liu et al 2022	N = 32<br>$\text{Female}_{proportion}$ = 0.69<br>Age = 20.5<br>BMI = NR Kg/m^2	CCT_{Cool} = 6500.0 K<br>CCT_{Warm} = 3000.0 K<br>CCT_{Fixed} = NR K<br>Light_{Bright} = NR lux<br>Light_{Dim} = NR lux<br>Light_{Fixed} = 250.0 lux	T_{Cool} = 23.0°C<br>T_{Warm} = 29.0 °C<br>T_{Fixed} = NR °C<br>RH = 40.0%<br>v_{air} = NR m/s<br>State = steady<br>Clo = 0.9	Effect on TSV = ✓<br>Effect TCV : ✓<br>Effect Phy = ✓
Wu et al 2022	N = 19<br>$\text{Female}_{proportion}$ = 0.47<br>Age = 22.75<br>BMI = 21.9 Kg/m^2	CCT_{Cool} = 6000.0 K<br>CCT_{Warm} = 1000.0 K<br>CCT_{Fixed} = NR K<br>Light_{Bright} = 1000.0 lux<br>Light_{Dim} = 200.0 lux<br>Light_{Fixed} = NR lux	T_{Cool} = 22.0°C<br>T_{Warm} = 30.0 °C<br>T_{Fixed} = NR °C<br>RH = 64.76%<br>v_{air} = 0.1 m/s<br>State = steady<br>Clo = 0.78	Effect on TSV = ✓<br>Effect TCV : ✓<br>Effect Phy = ✓
Lchner et al 2021	N = 61<br>$\text{Female}_{proportion}$ = 0.46<br>Age = 44.0<br>BMI = NR Kg/m^2	CCT_{Cool} = NR K<br>CCT_{Warm} = NR K<br>CCT_{Fixed} = NR K<br>Light_{Bright} = 1000.0 lux<br>Light_{Dim} = 300.0 lux<br>Light_{Fixed} = NR lux	T_{Cool} = 20.0°C<br>T_{Warm} = 30.0 °C<br>T_{Fixed} = NR °C<br>RH = NR%<br>v_{air} = NR m/s<br>State = steady<br>Clo = NR	Effect on TSV = NR<br>Effect TCV : ✓<br>Effect Phy = NR
Bellia et al 2021	N = 128<br>$\text{Female}_{proportion}$ = 0.50<br>Age = 23.1<br>BMI = NR Kg/m^2	CCT_{Cool} = 6000.0 K<br>CCT_{Warm} = 3000.0 K<br>CCT_{Fixed} = NR K<br>Light_{Bright} = NR lux<br>Light_{Dim} = NR lux<br>Light_{Fixed} = 300.0 lux	T_{Cool} = 20.0°C<br>T_{Warm} = 25.0 °C<br>T_{Fixed} = NR °C<br>RH = 52.7%<br>v_{air} = 0.02 m/s<br>State = steady	Effect on TSV = ✓<br>Effect TCV : ✗<br>Effect Phy = NR

Supplementary Table 1: Review of studies on the effect of light intensity and color on thermal sensation (TSV), thermal comfort (TCV), and physiological responses (Phy).

Study	Sample Characteristics	Light Conditions	Enviromental Conditions	Observed effects
			Clo = 0.71	
Kompier et al 2021	N = 23<br>$\text{Female}_{proportion}$ = 0.57<br>Age = 23.0<br>BMI = 22.0 Kg/m^2	CCT_{Cool} = 5890.0 K<br>CCT_{Warm} = 2676.0 K<br>CCT_{Fixed} = NR K<br>Light_{Bright} = 1012.0 lux<br>Light_{Dim} = 98.0 lux<br>Light_{Fixed} = NR lux	T_{Cool} = NR°C<br>T_{Warm} = NR °C<br>T_{Fixed} = 18.0 °C<br>RH = 67.4%<br>v_{air} = 0.01 m/s<br>State = steady<br>Clo = 0.7	Effect on TSV = ✗<br>Effect TCV : ✗<br>Effect Phy = ✗
Hangzi Wu et al 2020	N = 35<br>$\text{Female}_{proportion}$ = 0.23<br>Age = 23.91<br>BMI = 22.15 Kg/m^2	CCT_{Cool} = NR K<br>CCT_{Warm} = NR K<br>CCT_{Fixed} = 6500.0 K<br>Light_{Bright} = 1110.0 lux<br>Light_{Dim} = 180.0 lux<br>Light_{Fixed} = NR lux	T_{Cool} = 18.0°C<br>T_{Warm} = 28.0 °C<br>T_{Fixed} = NR °C<br>RH = 40.0%<br>v_{air} = 0.1 m/s<br>State = steady<br>Clo = 0.75	Effect on TSV = ✓<br>Effect TCV : NR<br>Effect Phy = NR
Kompier et al 2020	N = 38<br>$\text{Female}_{proportion}$ = 0.50<br>Age = 24.0<br>BMI = 22.0 Kg/m^2	CCT_{Cool} = 5854.0 K<br>CCT_{Warm} = 2708.0 K<br>CCT_{Fixed} = NR K<br>Light_{Bright} = 1021.0 lux<br>Light_{Dim} = 97.0 lux<br>Light_{Fixed} = NR lux	T_{Cool} = NR°C<br>T_{Warm} = NR °C<br>T_{Fixed} = 20.4 °C<br>RH = 47.2%<br>v_{air} = 0.04 m/s<br>State = steady<br>Clo = 0.7	Effect on TSV = ✗<br>Effect TCV : ✗<br>Effect Phy = ✗
Tsushima et al 2020	N = 118<br>$\text{Female}_{proportion}$ = 0.44<br>Age = 21.43<br>BMI = NR Kg/m^2	CCT_{Cool} = 5500.0 K<br>CCT_{Warm} = 3000.0 K<br>CCT_{Fixed} = NR K<br>Light_{Bright} = 300.0 lux	T_{Cool} = 24.0°C<br>T_{Warm} = 27.0 °C<br>T_{Fixed} = NR °C<br>RH = 50.0%	Effect on TSV = ✓<br>Effect TCV : NR

Supplementary Table 1: Review of studies on the effect of light intensity and color on thermal sensation (TSV), thermal comfort (TCV), and physiological responses (Phy).

Study	Sample Characteristics	Light Conditions	Enviromental Conditions	Observed effects
		Light_{Dim} = 80.0 lux<br>Light_{Fixed} = NR lux	v_{air} = NR m/s<br>State = steady<br>Clo = NR	Effect Phy = NR
Brambilla et al 2020	N = 37<br>$\text{Female}_{proportion}$ = 0.43<br>Age = 25.2<br>BMI = NR Kg/m^2	CCT_{Cool} = 6253.0 K<br>CCT_{Warm} = 2762.0 K<br>CCT_{Fixed} = NR K<br>Light_{Bright} = NR lux<br>Light_{Dim} = NR lux<br>Light_{Fixed} = 372.0 lux	T_{Cool} = 21.0°C<br>T_{Warm} = 26.0 °C<br>T_{Fixed} = NR °C<br>RH = 50.0%<br>v_{air} = NR m/s<br>State = steady<br>Clo = 0.78	Effect on TSV = ✓<br>Effect TCV : ✓<br>Effect Phy = NR
Chinazzo et al 2020	N = 57<br>$\text{Female}_{proportion}$ = 0.51<br>Age = 26.0<br>BMI = 21.6 Kg/m^2	CCT_{Cool} = NR K<br>CCT_{Warm} = NR K<br>CCT_{Fixed} = NR K<br>Light_{Bright} = NR lux<br>Light_{Dim} = NR lux<br>Light_{Fixed} = NR lux	T_{Cool} = 24.0°C<br>T_{Warm} = 29.0 °C<br>T_{Fixed} = NR °C<br>RH = 50.5%<br>v_{air} = 0.1 m/s<br>State = steady<br>Clo = NR	Effect on TSV = ✗<br>Effect TCV : ✓<br>Effect Phy = ✗
Alfano et al 2019	N = 81<br>$\text{Female}_{proportion}$ = 0.51<br>Age = 26.5<br>BMI = NR Kg/m^2	CCT_{Cool} = 6000.0 K<br>CCT_{Warm} = 3000.0 K<br>CCT_{Fixed} = NR K<br>Light_{Bright} = NR lux<br>Light_{Dim} = NR lux<br>Light_{Fixed} = 300.0 lux	T_{Cool} = NR°C<br>T_{Warm} = NR °C<br>T_{Fixed} = 20.3 °C<br>RH = 53.9%<br>v_{air} = 0.01 m/s<br>State = steady<br>Clo = 0.86	Effect on TSV = ✓<br>Effect TCV : NR<br>Effect Phy = NR
Chinazzo et al 2019	N = 84<br>$\text{Female}_{proportion}$ = 0.50	CCT_{Cool} = NR K<br>CCT_{Warm} = NR K	T_{Cool} = 19.0°C<br>T_{Warm} = 27.0 °C	Effect on TSV = ✗

Supplementary Table 1: Review of studies on the effect of light intensity and color on thermal sensation (TSV), thermal comfort (TCV), and physiological responses (Phy).

Study	Sample Characteristics	Light Conditions	Enviromental Conditions	Observed effects
	Age = 19.2<br>BMI = 21.2 Kg/m^2	CCT_{Fixed} = 6500.0 K<br>$Light_{Bright}$ = 1049.0 lux<br>$Light_{Dim}$ = 136.0 lux<br>$Light_{Fixed}$ = NR lux	T_{Fixed} = NR °C<br>RH = 49.45%<br>v_{air} = 0.01 m/s<br>State = steady<br>Clo = 0.7	Effect TCV : ✓<br>Effect Phy = ✗
Golasi et al 2019	N = 42<br>$Female_{proportion}$ = 0.43<br>Age = 23.25<br>BMI = 22.9 Kg/m^2	CCT_{Cool} = 11530.0 K<br>CCT_{Warm} = 1772.0 K<br>CCT_{Fixed} = NR K<br>$Light_{Bright}$ = NR lux<br>$Light_{Dim}$ = NR lux<br>$Light_{Fixed}$ = 510.0 lux	T_{Cool} = NR°C<br>T_{Warm} = NR °C<br>T_{Fixed} = 21.0 °C<br>RH = NR%<br>v_{air} = NR m/s<br>State = steady<br>Clo = 0.55	Effect on TSV = ✓<br>Effect TCV : ✓<br>Effect Phy = NR
Chinazzo et al 2018	N = 75<br>$Female_{proportion}$ = 0.40<br>Age = 19.7<br>BMI = 21.7 Kg/m^2	CCT_{Cool} = NR K<br>CCT_{Warm} = NR K<br>CCT_{Fixed} = NR K<br>$Light_{Bright}$ = NR lux<br>$Light_{Dim}$ = NR lux<br>$Light_{Fixed}$ = 541.0 lux	T_{Cool} = 19.0°C<br>T_{Warm} = 26.0 °C<br>T_{Fixed} = NR °C<br>RH = 34.65%<br>v_{air} = 0.1 m/s<br>State = steady<br>Clo = 0.7	Effect on TSV = ✓<br>Effect TCV : ✓<br>Effect Phy = ✗
Toftum et al 2018	N = 44<br>$Female_{proportion}$ = 0.36<br>Age = 23.8<br>BMI = 23.5 Kg/m^2	CCT_{Cool} = 6300.0 K<br>CCT_{Warm} = 2700.0 K<br>CCT_{Fixed} = NR K<br>$Light_{Bright}$ = NR lux<br>$Light_{Dim}$ = NR lux<br>$Light_{Fixed}$ = 1044.0 lux	T_{Cool} = 19.0°C<br>T_{Warm} = 27.0 °C<br>T_{Fixed} = NR °C<br>RH = 30.0%<br>v_{air} = 0.1 m/s<br>State = steady<br>Clo = 0.63	Effect on TSV = ✗<br>Effect TCV : ✓<br>Effect Phy = NR

Supplementary Table 1: Review of studies on the effect of light intensity and color on thermal sensation (TSV), thermal comfort (TCV), and physiological responses (Phy).

Study	Sample Characteristics	Light Conditions	Enviromental Conditions	Observed effects
Yang et al 2018	N = 120<br>$\text{Female}_{proportion}$ = 0.50<br>Age = 22.25<br>BMI = 21.75 Kg/m^2	CCT_{Cool} = NR K<br>CCT_{Warm} = NR K<br>CCT_{Fixed} = 6486.0 K<br>Light_{Bright} = 1000.0 lux<br>Light_{Dim} = 150.0 lux<br>Light_{Fixed} = NR lux	T_{Cool} = 20.0°C<br>T_{Warm} = 30.0 °C<br>T_{Fixed} = NR °C<br>RH = 45.0%<br>v_{air} = 0.1 m/s<br>State = steady<br>Clo = 0.75	Effect on TSV = ✓<br>Effect TCV : NR<br>Effect Phy = NR
Kulve et al 2017b	N = 16<br>$\text{Female}_{proportion}$ = 1.00<br>Age = 22.2<br>BMI = 21.5 Kg/m^2	CCT_{Cool} = NR K<br>CCT_{Warm} = NR K<br>CCT_{Fixed} = 4000.0 K<br>Light_{Bright} = 1200.0 lux<br>Light_{Dim} = 5.0 lux<br>Light_{Fixed} = NR lux	T_{Cool} = 26.0°C<br>T_{Warm} = 32.0 °C<br>T_{Fixed} = NR °C<br>RH = NR%<br>v_{air} = NR m/s<br>State = steady<br>Clo = 0.04	Effect on TSV = ✗<br>Effect TCV : ✗<br>Effect Phy = ✗
Kulve et al 2017a	N = 19<br>$\text{Female}_{proportion}$ = 1.00<br>Age = 22.3<br>BMI = 21.7 Kg/m^2	CCT_{Cool} = 5800.0 K<br>CCT_{Warm} = 2700.0 K<br>CCT_{Fixed} = NR K<br>Light_{Bright} = NR lux<br>Light_{Dim} = NR lux<br>Light_{Fixed} = 50.0 lux	T_{Cool} = 26.0°C<br>T_{Warm} = 32.0 °C<br>T_{Fixed} = NR °C<br>RH = NR%<br>v_{air} = NR m/s<br>State = steady<br>Clo = 0.04	Effect on TSV = ✗<br>Effect TCV : ✗<br>Effect Phy = ✗
Chou et al 2016	N = 8<br>$\text{Female}_{proportion}$ = 0.00<br>Age = 21.0<br>BMI = NR Kg/m^2	CCT_{Cool} = 6500.0 K<br>CCT_{Warm} = 3000.0 K<br>CCT_{Fixed} = NR K<br>Light_{Bright} = NR lux<br>Light_{Dim} = NR lux<br>Light_{Fixed} = 500.0 lux	T_{Cool} = 28.0°C<br>T_{Warm} = 30.0 °C<br>T_{Fixed} = NR °C<br>RH = 55.0%<br>v_{air} = 0.05 m/s<br>State = steady<br>Clo = NR	Effect on TSV = ✓<br>Effect TCV : ✗<br>Effect Phy = ✗

Supplementary Table 1: Review of studies on the effect of light intensity and color on thermal sensation (TSV), thermal comfort (TCV), and physiological responses (Phy).

Study	Sample Characteristics	Light Conditions	Enviromental Conditions	Observed effects
Huebner et al 2016a	N = 32<br>$Female_{proportion}$ = 0.44<br>Age = 24.7<br>BMI = 21.27 Kg/m^2	CCT_{Cool} = 6500.0 K<br>CCT_{Warm} = 2700.0 K<br>CCT_{Fixed} = NR K<br>$Light_{Bright}$ = NR lux<br>$Light_{Dim}$ = NR lux<br>$Light_{Fixed}$ = 522.5 lux	T_{Cool} = 20.0°C<br>T_{Warm} = 24.0 °C<br>T_{Fixed} = NR °C<br>RH = 50.0%<br>v_{air} = 0.1 m/s<br>State = dynamic<br>Clo = 0.695	Effect on TSV = ✓<br>Effect TCV : ✓<br>Effect Phy = NR
Baniya et al 2016	N = 16<br>$Female_{proportion}$ = 0.56<br>Age = 30.0<br>BMI = NR Kg/m^2	CCT_{Cool} = 6200.0 K<br>CCT_{Warm} = 2700.0 K<br>CCT_{Fixed} = NR K<br>$Light_{Bright}$ = NR lux<br>$Light_{Dim}$ = NR lux<br>$Light_{Fixed}$ = 500.0 lux	T_{Cool} = 20.0°C<br>T_{Warm} = 25.0 °C<br>T_{Fixed} = NR °C<br>RH = NR%<br>v_{air} = NR m/s<br>State = steady<br>Clo = 0.39	Effect on TSV = ✗<br>Effect TCV : ✗<br>Effect Phy = NR
Huebner et al 2016b	N = 32<br>$Female_{proportion}$ = 0.72<br>Age = 23.4<br>BMI = 21.68 Kg/m^2	CCT_{Cool} = 6500.0 K<br>CCT_{Warm} = 2700.0 K<br>CCT_{Fixed} = NR K<br>$Light_{Bright}$ = NR lux<br>$Light_{Dim}$ = NR lux<br>$Light_{Fixed}$ = 522.5 lux	T_{Cool} = 20.0°C<br>T_{Warm} = 24.0 °C<br>T_{Fixed} = NR °C<br>RH = 50.0%<br>v_{air} = 0.1 m/s<br>State = dynamic<br>Clo = 0.58	Effect on TSV = ✓<br>Effect TCV : ✗<br>Effect Phy = NR
Albers et al 2015	N = 199<br>$Female_{proportion}$ = 0.50<br>Age = 32.6<br>BMI = NR Kg/m^2	CCT_{Cool} = NR K<br>CCT_{Warm} = NR K<br>CCT_{Fixed} = NR K<br>$Light_{Bright}$ = NR lux<br>$Light_{Dim}$ = NR lux	T_{Cool} = 24.3°C<br>T_{Warm} = 32.1 °C<br>T_{Fixed} = NR °C<br>RH = 30.0%<br>v_{air} = 0.15 m/s	Effect on TSV = ✓<br>Effect TCV : ✓<br>Effect Phy = NR

Supplementary Table 1: Review of studies on the effect of light intensity and color on thermal sensation (TSV), thermal comfort (TCV), and physiological responses (Phy).

Study	Sample Characteristics	Light Conditions	Enviromental Conditions	Observed effects
		$Light_{Fixed}$ = NR lux	State = steady<br>Clo = 1.0	
Winzen et al 2014	N = 59<br>$Female_{proportion}$ = 0.31<br>Age = 21.78<br>BMI = NR Kg/m^2	CCT_{Cool} = NR K<br>CCT_{Warm} = NR K<br>CCT_{Fixed} = NR K<br>$Light_{Bright}$ = NR lux<br>$Light_{Dim}$ = NR lux<br>$Light_{Fixed}$ = NR lux	T_{Cool} = NR°C<br>T_{Warm} = NR °C<br>T_{Fixed} = 22.2 °C<br>RH = 62.0%<br>v_{air} = 0.01 m/s<br>State = steady<br>Clo = NR	Effect on TSV = ✓<br>Effect TCV : ✗<br>Effect Phy = NR
Ishibashi et 2010	N = 10<br>$Female_{proportion}$ = 0.00<br>Age = 23.9<br>BMI = 22.0 Kg/m^2	CCT_{Cool} = NR K<br>CCT_{Warm} = NR K<br>CCT_{Fixed} = 4578.0 K<br>$Light_{Bright}$ = 5000.0 lux<br>$Light_{Dim}$ = 30.0 lux<br>$Light_{Fixed}$ = NR lux	T_{Cool} = 15.0°C<br>T_{Warm} = 27.0 °C<br>T_{Fixed} = NR °C<br>RH = 50.0%<br>v_{air} = NR m/s<br>State = steady<br>Clo = 0.16	Effect on TSV = ✓<br>Effect TCV : NR<br>Effect Phy = ✓
Kim et al 2007	N = 9<br>$Female_{proportion}$ = 1.00<br>Age = 22.0<br>BMI = 19.77 Kg/m^2	CCT_{Cool} = NR K<br>CCT_{Warm} = NR K<br>CCT_{Fixed} = NR K<br>$Light_{Bright}$ = 3000.0 lux<br>$Light_{Dim}$ = 10.0 lux<br>$Light_{Fixed}$ = NR lux	T_{Cool} = 15.0°C<br>T_{Warm} = 30.0 °C<br>T_{Fixed} = NR °C<br>RH = 50.0%<br>v_{air} = NR m/s<br>State = dynamic<br>Clo = 0.21	Effect on TSV = ✓<br>Effect TCV : ✓<br>Effect Phy = ✓
Kim et al 2002	N = 7<br>$Female_{proportion}$ = 1.00<br>Age = 20.0	CCT_{Cool} = NR K<br>CCT_{Warm} = NR K<br>CCT_{Fixed} = NR K	T_{Cool} = 20.0°C<br>T_{Warm} = 26.0 °C<br>T_{Fixed} = NR °C	Effect on TSV = ✓<br>Effect TCV : NR

Supplementary Table 1: Review of studies on the effect of light intensity and color on thermal sensation (TSV), thermal comfort (TCV), and physiological responses (Phy).

Study	Sample Characteristics	Light Conditions	Enviromental Conditions	Observed effects
	BMI = 20.14 Kg/m^2	Light_{Bright} = 700.0 lux<br>Light_{Dim} = 70.0 lux<br>Light_{Fixed} = NR lux	RH = 55.0%<br>v_{air} = NR m/s<br>State = steady<br>Clo = 0.2	Effect Phy = ✓
Kim et al 2000	N = 5<br>$\text{Female}_{proportion}$ = 1.00<br>Age = 60.6<br>BMI = 24.4 Kg/m^2	CCT_{Cool} = NR K<br>CCT_{Warm} = NR K<br>CCT_{Fixed} = NR K<br>Light_{Bright} = 3000.0 lux<br>Light_{Dim} = 50.0 lux<br>Light_{Fixed} = NR lux	T_{Cool} = 15.0°C<br>T_{Warm} = 30.0 °C<br>T_{Fixed} = NR °C<br>RH = 50.0%<br>v_{air} = NR m/s<br>State = dynamic<br>Clo = 0.09	Effect on TSV = ✓<br>Effect TCV : ✓<br>Effect Phy = ✓
Teramoto et al 1996	N = 10<br>$\text{Female}_{proportion}$ = 1.00<br>Age = 20.1<br>BMI = 19.76 Kg/m^2	CCT_{Cool} = NR K<br>CCT_{Warm} = NR K<br>CCT_{Fixed} = NR K<br>Light_{Bright} = 4000.0 lux<br>Light_{Dim} = 200.0 lux<br>Light_{Fixed} = NR lux	T_{Cool} = 19.0°C<br>T_{Warm} = 31.0 °C<br>T_{Fixed} = NR °C<br>RH = 50.0%<br>v_{air} = 0.2 m/s<br>State = dynamic<br>Clo = 0.23	Effect on TSV = ✓<br>Effect TCV : NR<br>Effect Phy = ✓
Kim et al 1995	N = 7<br>$\text{Female}_{proportion}$ = 1.00<br>Age = 25.25<br>BMI = 18.88 Kg/m^2	CCT_{Cool} = NR K<br>CCT_{Warm} = NR K<br>CCT_{Fixed} = NR K<br>Light_{Bright} = 4000.0 lux<br>Light_{Dim} = 10.0 lux<br>Light_{Fixed} = NR lux	T_{Cool} = 15.0°C<br>T_{Warm} = 30.0 °C<br>T_{Fixed} = NR °C<br>RH = NR%<br>v_{air} = NR m/s<br>State = dynamic<br>Clo = 0.09	Effect on TSV = ✓<br>Effect TCV : ✗<br>Effect Phy = ✓

%

s.2

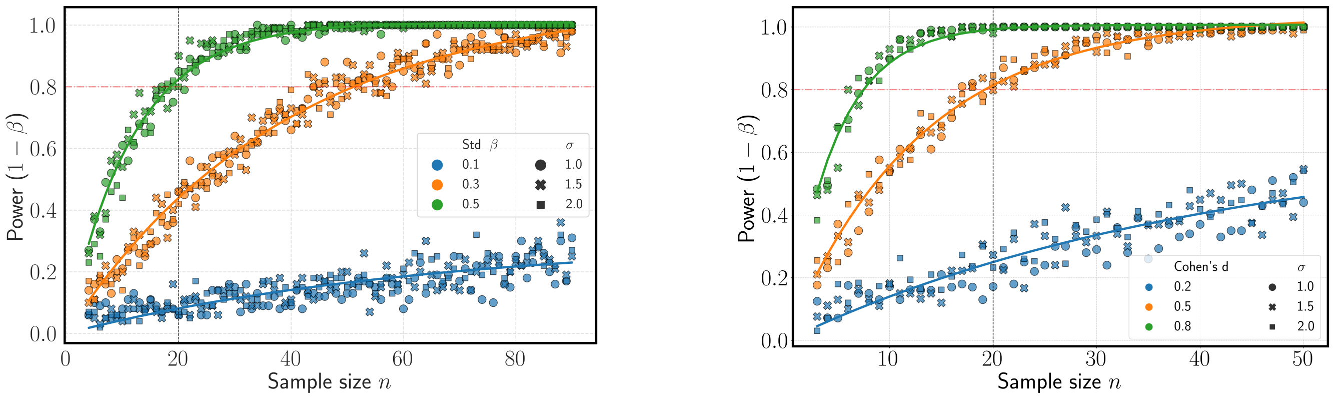

(a) **Power simulation using a Gaussian mixed-effects model with an identity link function.** Random intercept standard deviations were set to $\sigma = (1, 1.2, 2)$ and standardized regression coefficients to $\beta = (0.1, 0.3, 0.5)$. Each subject contributed 7 responses, with total sample sizes ranging from $N = 4$ to $N = 90$. For each combination of β, σ, and N, 50 simulation replicates were performed to estimate power.

(b) **Power Simulation using a mixed-effects cumulative model with logit link function:** ,Random intercept standard deviations were set to $\sigma = (1, 1.2, 2)$ and effect sizes (Cohen's d) to $(0.2, 0.5, 0.8)$. Each subject contributed 7 responses, with total sample sizes ranging from $N = 4$ to $N = 50$. For each combination of d, σ, and N, 50 simulation replicates were performed to estimate power.

Supplementary Figure 1: **Power Analysis Curves for (a) Continuous and (b) Ordinal Outcomes**

s.3

Supplementary Table 2: Thermal subjective evaluation scales [French Version]

**Métrique**	**Sensation thermique**	**Confort thermique**	**Plaisir thermique**	**Préférence thermique**
**Question**	Comment vous sentez-vous en ce moment précis?	Trouvez-vous cela...?	Considérez-vous cela?	Préféreriez-vous avoir...?
**Échelle**	**Réponses**	-	-	-
**-3**	Froid	Très Inconfortable	Très désagréable	
**-2**	Frais	Inconfortable	Désagréable	
**-1**	Légèrement Frais	Légèrement Inconfortable	Légèrement désagréable	Plus Froid
**0**	Neutre	Indifférent	Indifférent	Pas de changement
**+1**	Légèrement Chaud	Légèrement Confortable	Légèrement agréable	Plus Chaud
**+2**	Chaud	Confortable	Agréable	
**+3**	Très Chaud	Très Confortable	Très agréable	

Supplementary Table 3: Thermal Sensation Model Summary for the Steady-State Regime

**Model Fit**					
Formula	Sensation_thermique ∼ time_of_day * light * T + pmv_fanger + (1 + time_of_day * light | subject)				
Link	logit		Threshold	flexible	
LogLik	-429.52	AIC	911.03	NObs	523
**Random Effects**	Variance	Std. Dev.	Corr		
subject (Intercept)	9.378	3.062	-		
time_of_dayMID	2.847	1.687	-0.664		
lightBRI	5.637	2.374	-0.231, 0.397		
time_of_dayMID:lightBRI	5.236	2.288	0.323, -0.167, -0.794		
**Fixed Effects**	Estimate	Std. Error	z Value	P Value	
time_of_dayMID	0.36027	0.60540	0.595	(0.552)ns	
lightBRI	-0.78108	0.75151	-1.039	(0.299)ns	
T2	0.06569	0.45232	0.145	(0.885)ns	
T3	-0.82739	0.68213	-1.213	(0.225)ns	
pmv_fanger	1.05794	0.37612	2.813	(0.005)**	
time_of_dayMID:lightBRI	0.45372	0.80699	0.562	(0.574)ns	
time_of_dayMID:T2	-0.16021	0.59628	-0.269	(0.788)ns	
time_of_dayMID:T3	1.29458	0.88933	1.456	(0.145)ns	
lightBRI:T2	-1.90939	0.62308	-3.064	(0.002)**	
lightBRI:T3	-0.71985	0.88881	-0.810	(0.418)ns	
time_of_dayMID:lightBRI:T2	1.56908	0.85646	1.832	(0.067).	
time_of_dayMID:lightBRI:T3	0.34686	1.23151	0.282	(0.778)ns	
**Threshold Coefficients**	Estimate	Std. Error	z Value		
slightly cool|neutral	-7.1283	0.9303	-7.662		
neutral|slightly warm	-1.7314	0.7753	-2.233		
slightly warm|warm	2.6386	0.7888	3.345		
warm|hot	6.7720	0.9147	7.404		

Supplementary Table 4: Thermal Sensation Model Full Summary for Fan at Constant Speed Regime

**Model Fit**					
Formula	Sensation_thermique ∼ time_of_day * light + time_of_day * T + pmv_fanger + (1 + time_of_day * light | subject)				
Link	logit		Threshold	flexible	
LogLik	-452.76	AIC	947.52	NObs	673
**Random Effects**	Variance	Std. Dev.	Corr		
subject (Intercept)	21.337	4.619	-		
time_of_dayMID	3.143	1.773	-0.746		
lightBRI	4.370	2.090	-0.311, 0.714		
time_of_dayMID:lightBRI	4.568	2.137	0.394, -0.763, -0.940		
**Fixed Effects**	Estimate	Std. Error	z Value	P Value	
time_of_dayMID	0.27395	0.58045	0.472	(0.637)ns	
lightBRI	-0.42672	0.59418	-0.718	(0.473)ns	
T2	-0.20740	0.33211	-0.624	(0.532)ns	
T3	-1.14312	0.34972	-3.269	(0.001)**	
pmv_fanger	0.06170	0.13336	0.463	(0.644)ns	
time_of_dayMID:lightBRI	0.15105	0.65734	0.230	(0.818)ns	
time_of_dayMID:T2	-0.12568	0.46094	-0.273	(0.785)ns	
time_of_dayMID:T3	0.07186	0.47403	0.152	(0.880)ns	
**Threshold Coefficients**	Estimate	Std. Error	z Value		
cool|slightly cool	-3.796	1.132	-3.354		
slightly cool|neutral	1.348	1.117	1.207		
neutral|slightly warm	5.681	1.142	4.975		

s.4

Thermal sensation models full summary

Thermal Comfort models full summary

Skin temperature models full summary

Fan control model full summary

s.5

Posterior Distributions of Standardized Log-Odds Ratios in each condition

s.6

Supplementary Table 5: Thermal Sensation Model Full Summary for the Skin Rewarming Regime

**Model Fit**					
Formula	Sensation_thermique ∼ time_of_day * light + time_of_day * T + Day_Number + pmv_fanger + Gender + BSA + (1 + time_of_day * light | subject)				
Link	logit		Threshold	flexible	
LogLik	-931.98	AIC	1919.97	NObs	905
**Random Effects**	Variance	Std. Dev.	Corr		
subject (Intercept)	5.8914	2.4272	-		
time_of_dayMID	2.3558	1.5349	-0.803		
lightBRI	0.6383	0.7989	-0.732, 0.427		
time_of_dayMID:lightBRI	1.3141	1.1464	0.519, -0.774, -0.090		
**Fixed Effects**	Estimate	Std. Error	z Value	P Value	
time_of_dayMID	-0.008048	0.451612	-0.018	(0.986)ns	
lightBRI	-0.723881	0.283692	-2.552	(0.011)*	
T2	-0.368759	0.232196	-1.588	(0.112)ns	
T3	-0.685542	0.243519	-2.815	(0.005)**	
Day_Number2	-0.431399	0.378838	-1.139	(0.255)ns	
Day_Number3	-0.452392	0.298827	-1.514	(0.130)ns	
Day_Number4	-0.861516	0.354663	-2.429	(0.015)*	
pmv_fanger	0.802373	0.154926	5.179	(2.23e-07)***	
GenderM	-2.123052	0.944773	-2.247	(0.025)*	
BSA	1.506155	0.537226	2.804	(0.005)**	
time_of_dayMID:lightBRI	0.606988	0.379530	1.599	(0.110)ns	
time_of_dayMID:T2	0.210895	0.328383	0.642	(0.521)ns	
time_of_dayMID:T3	0.419114	0.332443	1.261	(0.207)ns	
**Threshold Coefficients**	Estimate	Std. Error	z Value		
cool|slightly cool	-8.1083	0.8711	-9.308		
slightly cool|neutral	-6.1942	0.8214	-7.541		
neutral|slightly warm	-3.1071	0.7960	-3.904		
slightly warm|warm	0.2406	0.7881	0.305		
warm|hot	3.5064	0.8182	4.286		

Supplementary Table 6: Thermal Comfort Model Full Summary for the Steady-State Regime

**Model Fit**					
Formula	Confort_thermique ∼ time_of_day * light + time_of_day * T + pmv_fanger + (1 + time_of_day * light | subject)				
Link	logit		Threshold	flexible	
LogLik	-584.17	AIC	1214.34	NObs	521
**Random Effects**	Variance	Std. Dev.	Corr		
subject (Intercept)	8.260	2.874	-		
time_of_dayMID	9.637	3.104	-0.392		
lightBRI	5.636	2.374	-0.204, 0.870		
time_of_dayMID:lightBRI	18.161	4.262	0.167, -0.818, -0.829		
**Fixed Effects**	Estimate	Std. Error	z Value	P Value	
time_of_dayMID	0.8422	0.8035	1.048	(0.295)ns	
lightBRI	1.3340	0.6593	2.023	(0.043)*	
T2	0.8924	0.2933	3.042	(0.002)**	
T3	0.9113	0.4279	2.130	(0.033)*	
pmv_fanger	-0.7817	0.3200	-2.443	(0.015)*	
time_of_dayMID:lightBRI	-1.4008	1.0529	-1.330	(0.183)ns	
time_of_dayMID:T2	-1.1303	0.3865	-2.924	(0.003)**	
time_of_dayMID:T3	-0.1806	0.5465	-0.330	(0.741)ns	
**Threshold Coefficients**	Estimate	Std. Error	z Value		
Uncomfortable|Slightly Uncomfortable	-3.38837	0.72721	-4.659		
Slightly Uncomfortable|Indifferent	0.01622	0.69991	0.023		
Indifferent|Slightly Comfortable	2.68079	0.71307	3.759		
Slightly Comfortable|Comfortable	5.43445	0.75897	7.160		
Comfortable|Very Comfortable	8.42302	0.89799	9.380		

Supplementary Table 7: Thermal Comfort Model Full Summary for Fan at Constant Speed Regime

**Model Fit**				
Formula	Confort_thermique ∼ time_of_day * light + light * T + time_of_day * T + pmv_fanger + BSA + BMI + Gender + (1 + time_of_day * light | subject)			
Link	logit		Threshold	flexible
LogLik	-584.30	AIC	1222.60	NObs 672
**Random Effects**	Variance	Std. Dev.	Corr	
subject (Intercept)	17.368	4.168	-	
time_of_dayMID	5.688	2.385	-0.772	
lightBRI	7.353	2.712	0.127, 0.465	
time_of_dayMID:lightBRI	14.092	3.754	-0.414, -0.235, -0.715	
**Fixed Effects**	Estimate	Std. Error	z Value	P Value
time_of_dayMID	0.7726	0.6691	1.155	(0.248)ns
lightBRI	0.8833	0.7289	1.212	(0.226)ns
T2	1.0004	0.3764	2.658	(0.008)**
T3	1.7529	0.3955	4.432	(9.32e-06)***
pmv_fanger	0.1393	0.1237	1.126	(0.260)ns
BSA	-2.5482	0.4885	-5.216	(1.83e-07)***
BMI	2.0237	0.3385	5.979	(2.24e-09)***
GenderM	0.4584	0.7687	0.596	(0.551)ns
time_of_dayMID:lightBRI	0.2188	0.9466	0.231	(0.817)ns
lightBRI:T2	-1.0818	0.4345	-2.490	(0.013)*
lightBRI:T3	-1.1082	0.4374	-2.533	(0.011)*
time_of_dayMID:T2	-0.7239	0.4256	-1.701	(0.089).
time_of_dayMID:T3	-1.0560	0.4341	-2.433	(0.015)*
**Threshold Coefficients**	Estimate	Std. Error	z Value	
Slightly Uncomfortable|Indifferent	-3.838	1.104	-3.477	
Indifferent|Slightly Comfortable	-1.720	1.093	-1.573	
Slightly Comfortable|Comfortable	1.335	1.091	1.224	
Comfortable|Very Comfortable	6.942	1.146	6.056	

Supplementary Table 8: Thermal Comfort Model Full Summary for Fan Off Regime

**Model Fit**				
Formula	Confort_thermique ∼ light * time_of_day + T + Day_Number + pmv_fanger + Weight_ + (1 + time_of_day * light | subject)			
Link	logit		Threshold	flexible
LogLik	-1024.57	AIC	2099.14	NObs 902
**Random Effects**	Variance	Std. Dev.	Corr	
subject (Intercept)	4.848	2.202	-	
time_of_dayMID	4.234	2.058	-0.397	
lightBRI	2.978	1.726	-0.053, 0.904	
time_of_dayMID:lightBRI	7.560	2.750	0.090, -0.804, -0.804	
**Fixed Effects**	Estimate	Std. Error	z Value	P Value
lightBRI	1.0307	0.4522	2.279	(0.023)*
time_of_dayMID	0.0585	0.5115	0.114	(0.909)ns
T2	0.2547	0.1590	1.602	(0.109)ns
T3	0.4631	0.1675	2.765	(0.006)**
Day_Number2	0.6004	0.3333	1.801	(0.072).
Day_Number3	0.7377	0.3931	1.877	(0.061).
Day_Number4	0.9699	0.4293	2.259	(0.024)*
pmv_fanger	-0.7540	0.1479	-5.097	(3.45e-07)***
Weight_	-1.1363	0.4476	-2.538	(0.011)*
lightBRI:time_of_dayMID	-0.9460	0.6883	-1.374	(0.169)ns
**Threshold Coefficients**	Estimate	Std. Error	z Value	
Uncomfortable|Slightly Uncomfortable	-2.7821	0.5938	-4.686	
Slightly Uncomfortable|Indifferent	0.2070	0.5857	0.353	
Indifferent|Slightly Comfortable	2.3246	0.5929	3.921	
Slightly Comfortable|Comfortable	4.9241	0.6180	7.967	
Comfortable|Very Comfortable	8.3393	0.7440	11.209	

Supplementary Table 9: Mean Skin Temperature Linear Mixed-Effects Model Full Summary for Steady State Regime

**Model Fit**				
Formula	MST_Ramanathan ∼ time_of_day * light * T + pmv_fanger + (1 + time_of_day * light | subject)			
Method	REML		Residual Std. Dev.	0.245
REML Criterion	540.7		Observations	2809
**Random Effects**	Variance	Std. Dev.	Corr	
subject (Intercept)	0.763	0.874	-	
time_of_dayMID	0.536	0.732	-0.50	
lightBRI	0.507	0.712	-0.76, 0.51	
time_of_dayMID:lightBRI	1.094	1.046	0.41, -0.52, -0.51	
**Fixed Effects**	Estimate	Std. Error	t Value	P value
(Intercept)	-0.4079	0.2009	-2.031	(0.057).
time_of_dayMID	0.7417	0.1696	4.375	(0.000)***
lightBRI	-0.4057	0.1656	-2.450	(0.024)*
T2	0.1578	0.0216	7.325	(3.1e-13)***
T3	0.1810	0.0599	3.020	(0.003)**
pmv_fanger	0.2993	0.0228	13.133	(<2e-16)***
time_of_dayMID:lightBRI	0.5781	0.2417	2.391	(0.028)*
time_of_dayMID:T2	0.0010	0.0271	0.036	(0.971)ns
time_of_dayMID:T3	-0.0328	0.0824	-0.398	(0.691)ns
lightBRI:T2	0.2135	0.0268	7.981	(2.1e-15)***
lightBRI:T3	0.1184	0.0820	1.444	(0.149)ns
time_of_dayMID:lightBRI:T2	-0.3970	0.0378	-10.503	(<2e-16)***
time_of_dayMID:lightBRI:T3	-0.3006	0.1160	-2.591	(0.010)*

Supplementary Table 10: Mean Skin Temperature Linear Mixed-Effects Model Full Summary for Fan Constant Speed Regime

**Model Fit**				
Formula	MST_ISO ∼ time_of_day * light * T + pmv_fanger + Gender + (1 + time_of_day * light | subject)			
Method	REML		Residual Std. Dev.	0.422
REML Criterion	1560.8		Observations	1139
**Random Effects**	Variance	Std. Dev.	Corr	
subject (Intercept)	0.687	0.829	-	
time_of_dayMID	0.539	0.734	-0.48	
lightBRI	0.159	0.399	-0.66, 0.51	
time_of_dayMID:lightBRI	1.211	1.100	0.13, -0.61, -0.42	
**Fixed Effects**	Estimate	Std. Error	t Value	P value
(Intercept)	-0.1104	0.2391	-0.462	(0.649)ns
time_of_dayMID	0.5050	0.1797	2.810	(0.010)*
lightBRI	-0.3508	0.1102	-3.184	(0.003)**
T2	0.2335	0.0614	3.802	(0.000)***
T3	0.2382	0.0625	3.813	(0.000)***
pmv_fanger	0.1340	0.0192	6.966	(5.6e-12)***
GenderM	-0.5308	0.2629	-2.019	(0.060).
time_of_dayMID:lightBRI	0.8961	0.2670	3.357	(0.003)**
time_of_dayMID:T2	0.0039	0.0867	0.045	(0.964)ns
time_of_dayMID:T3	-0.1734	0.0870	-1.995	(0.046)*
lightBRI:T2	0.1360	0.0875	1.554	(0.121)ns
lightBRI:T3	0.2997	0.0874	3.428	(0.001)***
time_of_dayMID:lightBRI:T2	-0.6143	0.1225	-5.012	(6.3e-07)***
time_of_dayMID:lightBRI:T3	-0.7352	0.1226	-5.999	(2.7e-09)***

Supplementary Table 11: Mean Skin Temperature Linear Mixed-Effects Model Full Summary for Skin Rewarming Regime

**Model Fit**				
Formula	MST_ISO ∼ time_of_day * light * T + pmv_fanger + BSA + (1 + time_of_day * light | subject)			
Method	REML		Residual Std. Dev.	0.365
REML Criterion	1722.7		Observations	1671
**Random Effects**	Variance	Std. Dev.	Corr	
subject (Intercept)	0.516	0.719	-	
time_of_dayMID	0.520	0.721	-0.49	
lightBRI	0.226	0.475	-0.63, 0.45	
time_of_dayMID:lightBRI	0.970	0.985	0.23, -0.62, -0.36	
**Fixed Effects**	Estimate	Std. Error	t Value	P value
(Intercept)	-0.4337	0.1675	-2.588	(0.019)*
time_of_dayMID	0.8257	0.1713	4.821	(0.000)***
lightBRI	-0.3944	0.1173	-3.363	(0.003)**
T2	0.2553	0.0432	5.912	(4.1e-09)***
T3	0.3979	0.0487	8.166	(6.4e-16)***
pmv_fanger	0.3640	0.0247	14.749	(<2e-16)***
BSA	-0.3564	0.1196	-2.981	(0.008)**
time_of_dayMID:lightBRI	0.6311	0.2337	2.700	(0.014)*
time_of_dayMID:T2	-0.2083	0.0597	-3.489	(0.000)***
time_of_dayMID:T3	-0.3668	0.0650	-5.648	(1.9e-08)***
lightBRI:T2	0.0251	0.0610	0.412	(0.681)ns
lightBRI:T3	0.2065	0.0650	3.177	(0.002)**
time_of_dayMID:lightBRI:T2	-0.4661	0.0839	-5.555	(3.3e-08)***
time_of_dayMID:lightBRI:T3	-0.6562	0.0906	-7.243	(6.8e-13)***

Supplementary Table 12: Fan control Linear Mixed-Effects Model Full Summary

**Model Fit**				
Formula	Fan_Control ∼ time_of_day * light * Fan_T + Temp_avg + Gender + Chrono + Day_Number + BSA + BMI + (1 + time_of_day * light | subject)			
Method	REML		Residual Std. Dev.	0.393
REML Criterion	1529.6		Observations	1296
**Random Effects**	Variance	Std. Dev.	Corr	
subject (Intercept)	0.488	0.699	-	
time_of_dayMID	0.363	0.603	0.31	
lightBRI	0.251	0.501	0.10, 0.79	
time_of_dayMID:lightBRI	0.794	0.891	0.11, -0.87, -0.71	
**Fixed Effects**	Estimate	Std. Error	t Value	P value
(Intercept)	-0.4992	0.3038	-1.643	(0.118)ns
time_of_dayMID	-0.1247	0.1539	-0.810	(0.427)ns
lightBRI	-0.2412	0.1306	-1.847	(0.080).
Fan_T2	-0.2141	0.0546	-3.921	(9.3e-05)***
Fan_T3	-0.0356	0.0591	-0.603	(0.547)ns
Temp_avg	0.1573	0.0288	5.456	(1.3e-07)***
GenderM	0.7487	0.3216	2.328	(0.038)*
Chronomoderate late	-0.1235	0.2808	-0.440	(0.667)ns
ChronoSlight late	1.0050	0.2977	3.376	(0.006)**
Day_Number2	-0.1702	0.0880	-1.934	(0.065).
Day_Number3	-0.0216	0.1242	-0.173	(0.864)ns
Day_Number4	-0.2014	0.1084	-1.858	(0.077).
BSA	-0.2806	0.2085	-1.346	(0.204)ns
BMI	-0.0309	0.1531	-0.202	(0.844)ns
time_of_dayMID:lightBRI	0.3674	0.2257	1.628	(0.120)ns
time_of_dayMID:Fan_T2	0.3073	0.0760	4.044	(5.6e-05)***
time_of_dayMID:Fan_T3	0.1289	0.0772	1.669	(0.095).
lightBRI:Fan_T2	0.2521	0.0765	3.296	(0.001)**
lightBRI:Fan_T3	0.0047	0.0761	0.062	(0.951)ns
time_of_dayMID:lightBRI:Fan_T2	-0.2587	0.1072	-2.413	(0.016)*
time_of_dayMID:lightBRI:Fan_T3	0.1161	0.1080	1.075	(0.282)ns

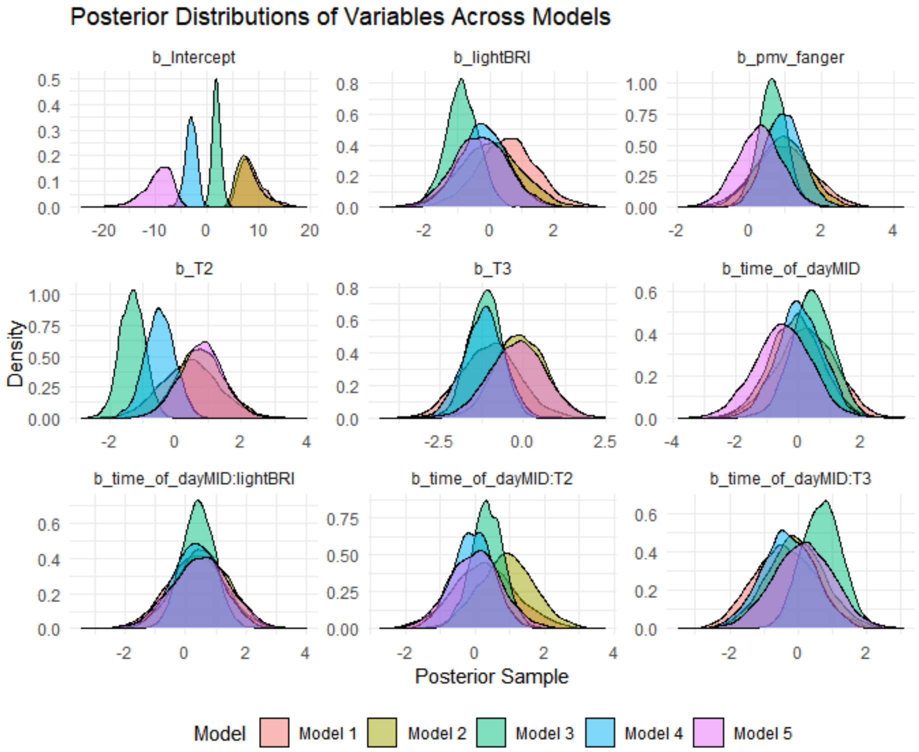

Supplementary Figure 2: Posterior Distributions of Standardized Log-Odds Ratios for Thermal Comfort Predictors Across Adjacent Response Categories in Bayesian Binary Logistic Models (Steady-State Conditions)

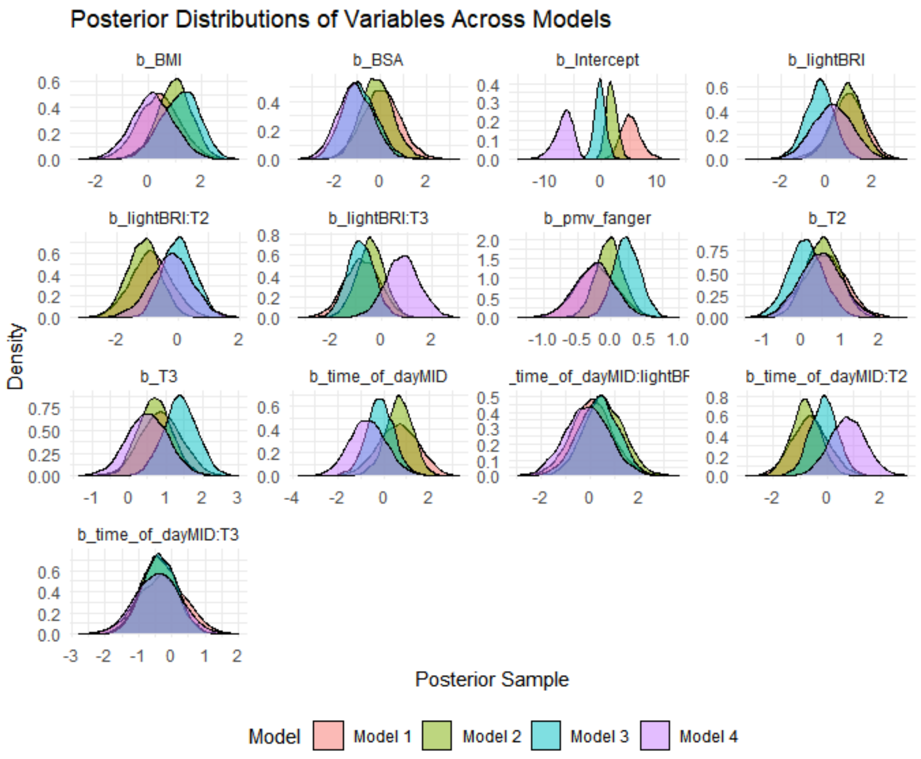

Supplementary Figure 3: Posterior Distributions of Standardized Log-Odds Ratios for Thermal Comfort Predictors Across Adjacent Response Categories in Bayesian Binary Logistic Models (Fan constant speed)

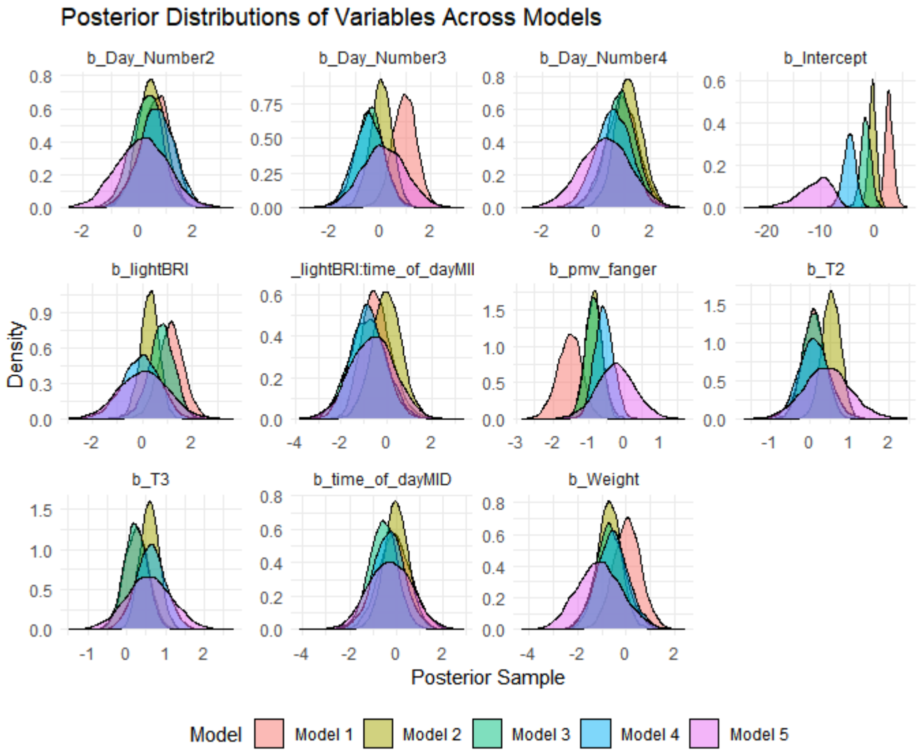

Supplementary Figure 4: Posterior Distributions of Standardized Log-Odds Ratios for Thermal Comfort Predictors Across Adjacent Response Categories in Bayesian Binary Logistic Models (Skin rewarming)

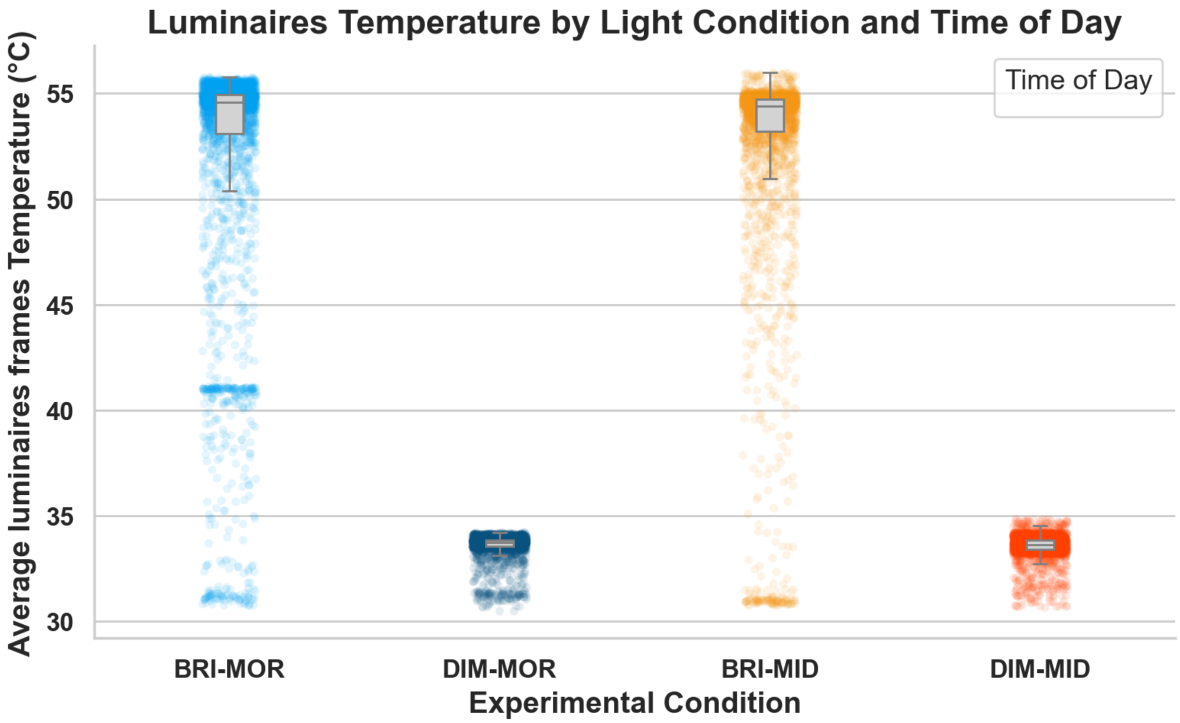

Supplementary Figure 5: Average measured frame temperature of the 4 × 4 LED panels under each lighting setup.
